# Leveraging interindividual variability of regulatory activity for refining genetic regulation of gene expression in schizophrenia

**DOI:** 10.1038/s41380-022-01768-4

**Published:** 2022-09-16

**Authors:** Maris Alver, Nikolaos Lykoskoufis, Anna Ramisch, Emmanouil T. Dermitzakis, Halit Ongen

**Affiliations:** 1 Department of Genetic Medicine and Development, University of Geneva, Geneva, Switzerland; 2 Swiss Institute of Bioinformatics, University of Geneva, Geneva, Switzerland; 3 Institute of Genetics and Genomics in Geneva, University of Geneva, Geneva, Switzerland; 4 Estonian Genome Center, Institute of Genomics, University of Tartu, Tartu, Estonia

**Keywords:** Genetics, Molecular biology, Schizophrenia, Neuroscience

## Abstract

Schizophrenia is a polygenic psychiatric disorder with limited understanding about the mechanistic changes in gene expression regulation. To elucidate on this, we integrate interindividual variability of regulatory activity (ChIP-sequencing for H3K27ac histone mark) with gene expression and genotype data captured from the prefrontal cortex of 272 cases and controls. By measuring interindividual correlation among proximal chromatin peaks, we show that regulatory element activity is structured into 10,936 and 10,376 cis-regulatory domains in cases and controls, respectively. The schizophrenia-specific cis-regulatory domains are enriched for fetal-specific (*p* = 0.0014, OR = 1.52) and depleted of adult-specific regulatory activity (*p* = 3.04 × 10^−50^, OR = 0.57) and are enriched for SCZ heritability (*p* = 0.001). By studying the interplay among genetic variants, gene expression, and cis-regulatory domains, we ascertain that changes in coordinated regulatory activity tag alterations in gene expression levels (*p* = 3.43 × 10^−5^, OR = 1.65), unveil case-specific QTL effects, and identify regulatory machinery changes for genes affecting synaptic function and dendritic spine morphology in schizophrenia. Altogether, we show that accounting for coordinated regulatory activity provides a novel mechanistic approach to reduce the search space for unveiling genetically perturbed regulation of gene expression in schizophrenia.

## Introduction

Schizophrenia (SCZ) is a severe mental illness that oftentimes leads to a lifetime of chronic disability. While several lines of evidence converge on the neurodevelopmental origin for the disorder and augment the impact of both genetic and environmental factors in disease aetiology, the pathophysiology of SCZ remains incompletely understood with little progress in novel treatment development [1, 2].

The last decades of extensive research have yielded valuable insights into the genomic and molecular underpinnings of SCZ. Large-scale genomic analyses triggered by prior studies of strong heritability estimates for SCZ (60–80%) [3, 4] have unveiled its highly polygenic architecture [5–7]. Complemented by gene expression and chromatin profiling analyses from dorsolateral prefrontal cortex (DLPFC), the SCZ risk variants have been shown to localize in functional regulatory genomic elements with approximately half of these displaying brain tissue-specific expression quantitative trait locus (eQTL) effects [5, 8–10], and to be enriched for open chromatin and evolutionary conserved regions [11, 12]. Genes identified by differential expression and genome-wide association analyses (GWAS) are associated with brain developmental pathways, synaptic function, and immune response [13–15]. While immense efforts have resulted in comprehensive SCZ-specific resources for perturbed gene expression and altered chromatin patterns and for fine-mapping and annotating discovered disease-associated genomic associations, little is known about the regulatory machinery changes and how genetic effects are propagated onto gene expression that drive molecular abnormalities in SCZ development.

Gene transcription profiles are defined by the activity of regulatory elements (REs) that overlap with open chromatin. Since the activity of REs are modulated by genetic variation, changes in chromatin accessibility result in gene expression variability and hence constitute as an intermediate phenotype for profiling eQTL effects on gene expression [16–20]. Systematic measurement of interindividual correlation between chromatin activity levels has revealed that the variability of nearby regulatory activity is structured into well-delimited sets of cis-regulatory domains (CRDs) [21]. The coordinated activity of REs within CRDs are under tight genetic control, mediate cis and trans effects of genetic variants onto gene expression, constitute finer organization within topologically associated domains (TADs), and provide a higher-order structural resolution of functional regulatory associations [21]. Accounting for the three-dimensional (3D) genome organization in cis that captures concerted effect of regulatory activity could thereby facilitate more robust signal detection for identifying disruption in regulatory function and for delineating deviations in gene expression cascades specific to disease. For instance, Girdhar et al. reported that correlated hyper-acetylated histone peaks are enriched for regulatory regions linked to excitatory neurons, SCZ heritability, and development, align with nuclear topography, and are associated with genes that are differentially expressed in SCZ [22]. To extend these findings and characterize genetically perturbed regulatory machinery changes specific to SCZ, we set out to analyse the interplay among genetic variants, coordinated regulatory activity and gene expression. To this end, we integrated genome-wide genotyping data with RE activity levels (chromatin immunoprecipitation sequencing (ChIP-sequencing) profiled for histone mark H3K27ac) and transcriptomic profiles (bulk RNA-sequencing) obtained from the DLPFC of SCZ cases and control subjects within the Human Brain Collection Core (HBCC; dbGaP phs000979.v3.p2) (Supplementary Fig. 1). At least two levels of molecular data were available for 272 individuals: 98 SCZ cases and 174 controls (188 males and 84 females, 164 African Americans and 108 Europeans; Supplementary Fig. 2).

## Materials and methods

### Molecular and phenotype data

Molecular and phenotype data for HBCC was accessed through dbGaP (study accession phs000979.v3.p2; request #88083-1 approved by NIH on January 31st, 2020). All patients met DSM-IV criteria for a lifetime Axis I diagnosis of psychiatric disorders including schizophrenia or schizoaffective disorder. Controls had no history of psychiatric diagnoses or addictions. For genotype data, we determined the intersect of single nucleotide variant (SNV) content across three Illumina genotyping arrays (HumanHap650Y, Human1M-Duov3 and HumanOmni5M-Quad) after filtering the SNVs using standard procedure with PLINK v2.0 [23] and imputed the derived genotype matrix using the TOPMed Imputation Reference panel [24]. Next, we applied post-imputation quality control filters by European and African American ancestral group separately and considered the union of filtered SNVs retrieved in both ancestry sets for the final SNV set. This yielded 9,516,522 SNVs in 272 individuals. Sequence data was mapped onto the human genome (hg19) with either BWA-MEM v0.7.16 [25] for ChIP-sequencing data or STAR v2.7 [26] for RNA-sequencing data. Gene expression was quantified using QTLtools [27] and filtered for protein-coding and lincRNA genes and for the union quantifications detected in ≥50% in SCZ cases and in ≥50% in controls. This yielded 21,988 genes for 243 individuals. ChIP-sequencing peak calling and quantification was carried out with HOMER v14.11.1 [28]. We first determined ChIP-sequencing peak coordinates across SCZ cases and controls to get a population scale call set of ChIP-sequencing peaks and then quantified the peaks for each individual according to the identified peak coordinates. This yielded 141,218 ChIP-sequencing peaks for 193 individuals. To account for confounding factors in gene expression and ChIP-sequencing peak data, we regressed out ancestry (captured by principal component (PC) analysis on genotype data), and technical variables (captured by PC analysis on molecular phenotype data). For the latter, we used the number of PCs that maximized the number of QTL discovery. Both gene expression and ChIP-sequencing data were normalized such that these matched a normal distribution with mean 0 and standard deviation 1.

### Cis-regulatory domain calling and quantification

For CRD calling, we used the pipeline developed in Delaneau et al. [21]. First, we built a correlation map from chromatin data by systematically measuring interindividual correlation (i.e., Pearson correlation coefficient) between all possible pairs of ChIP-sequencing peak quantifications located on the same chromosome (within a 250-peak sliding window). Next, we applied hierarchical clustering on the data on a per chromosome basis to get a binary tree that regrouped chromatin peaks for each chromosome depending on the correlation levels they exhibited. We relied on three empirical criteria for CRD calling: (i) overall correlation that required the mean level of correlation within a CRD to be at least twice the background, (ii) edge correlation that required the mean level of correlation of the peaks at the CRD boundaries to be at least twice the background, and (iii) a requirement that the CRD covered at least two non-overlapping regulatory elements. We quantified CRD activity on per-individual basis by enumerating all ChIP-sequencing peaks per CRD and taking the mean of all single peak quantifications per individual to retrieve a single quantification value for each individual. CRDs called in SCZ cases were used for characterizing SCZ-specific CRDs. CRDs identified in the combined set were used for the rest of the downstream analyses.

### CRD structure analysis

To assess the features of SCZ-specific CRDs, we considered only CRDs in SCZ cases composed of peaks not regrouping into any CRD in controls. For peak activity estimation, we used ChIP-sequencing peak quantifications uncorrected for covariates and applied Mann-Whitney U test per peak activity between SCZ cases and controls. Significant differences between SCZ cases and controls were determined at false discovery rate (FDR) 5% using R/*qvalue* package [29]. For estimating peak activity correlation per SCZ-specific CRDs between SCZ cases and controls, we used ChIP-sequencing peak quantifications corrected for biological and technical covariates, calculated the mean Pearson correlation estimate between peak activities per CRD and used Mann-Whitney U test for comparing these correlation estimates between SCZ cases and controls.

### Differential CRD activity and differential gene expression analysis

Differential CRD activity analysis and differential gene expression analysis were carried out using DESeq2 [30]. Significant associations were determined at FDR 5% [29]. For differential CRD activity analysis, we used ChiP-sequencing peak counts obtained with HOMER [28] and summed these up per CRD using the correlation map identified in the combined set. For differential gene expression analysis, we used RNA-sequencing read counts. To identify covariates for correction, we carried out association testing (i) between all available biological and technical covariates and disease status (SCZ/control) (Mann-Whitney U test), and (ii) between all available biological and technical covariates and individual ChIP-sequencing peak activity or gene expression quantifications (linear regression) and calculated π1 estimate [29] to identify the proportion of true associations. R*/clusterProfiler* package [31] was used for gene set enrichment analysis.

### Enrichment analysis of CRD peaks for SCZ GWAS variants

Both MAGMA v1.10 [32] and partitioned LD score regression v1.0.1 [33] were used to test SCZ GWAS variant [7] enrichment in ChIP-seq peaks regrouping into SCZ-specific CRDs and into significantly differentially active CRDs.

### Mapping molecular quantitative trait loci (QTLs)

QTL mapping was carried out using the standard procedure implemented in the QTLtools software package [27]. Specifically, we performed 1,000 permutations to correct for the number of genetic variants being tested in cis per molecular phenotype (+/− 1 Mb window) and corrected for the number of molecular phenotypes being tested genome-wide using FDR [29]. To identify multiple QTLs with independent effects on a molecular phenotype, we used the conditional analysis approach based on a forward-backward scan implemented in QTLtools [27]. For SCZ-specific QTL discovery, we considered QTL effects identified in SCZ cases and for each variant-phenotype pair ran a linear regression including genotype, disease status (SCZ/control), and covariates, and tested for significance of a genotype × disease status interaction on the molecular phenotype (gene expression or CRD activity). This was followed with FDR 5% correction for the number of QTLs tested. We assessed the likelihood of a shared functional effect between SCZ risk variants from four GWAS studies [5–7, 34] and SCZ-identified QTLs using regulatory trait concordance (RTC) [35, 36].

### CRD and gene association

We used QTLtools cis permuation pass [27] to identify CRDs associated with a gene in a +/−1 Mb window from a transcription start site of a gene. We performed these analyses (i) to identify genes associated with SCZ-specific CRDs using CRDs identified in SCZ cases, and (ii) to capture comparable associations for SCZ cases and controls using CRDs detected in the combined set. We tested QTL effects for association with the other molecular phenotype (i.e., aCRD-QTLs with gene expression and eQTLs with CRD activity) via CRD-gene associations detected across all samples at nominal significance.

### Causal inference

To quantify gene-CRD pairs identified as significant at FDR 5% across SCZ cases and controls, we used PC analysis-based dimensionality reduction. For each gene-CRD pair, we aggregated gene expression with CRD activity and used the coordinates on PC1 as new pseudo-phenotypes for QTL mapping in a cis window. This effectively gave us eCRDQTL-CRD-gene triplets consisting of a genetic variant, a CRD, and a gene, all associated with each other.

We applied a Bayesian Network approach to infer the most likely causal relationship for eCRDQTL-CRD-gene triplets common to SCZ cases and controls and conducted the analyses separately in SCZ cases and in controls. This approach allowed to estimate the most likely network from which the observed data originates by calculating the posterior probabilities for the three possible causal models: [37] (i) causal model in which the genetic variant affects first the CRD and then the gene, (ii) reactive model in which the genetic variant affects the gene and then the CRD, (iii) independent model in which the genetic variant affects the gene and the CRD independently.

To provide confidence for retrieved probabilities, we carried out 100 bootstrapping runs for each tested triplet separately for SCZ cases and controls using sampling with replacement. We estimated how many times the most probable model across bootstrapping runs for each triplet was the same as in the original Bayesian Network results and filtered out all triplets that fell below a confidence threshold of 55%: this corresponds to the lower quartile value in SCZ cases. R*/clusterProfiler* package [31] was used for gene set enrichment analysis for genes that belonged to triplets showing directional change from eCRD-QTL onto gene expression/CRD activity between SCZ cases and controls. We considered two scenarios: (i) causal model in controls, but reactive/independent in SCZ cases; (ii) reactive/independent in controls, but causal in SCZ cases.

Extended methods are provided in the Supplementary.

## Results

### Distinct regulatory element coordination in SCZ

To study the coordination of REs, we systematically measured interindividual correlation between nearby chromatin peaks. We identified 10,938 CRDs in SCZ cases, 10,376 CRDs in controls and 11,374 CRDs in the combined set (i.e., across SCZ cases and controls), regrouping 28.9% (*n* = 40,819), 31.4% (*n* = 44,391) and 38.4% (*n* = 54,278) of the peaks, respectively (Table 1, Supplementary Table 1, Supplementary Table 2), and capturing a higher-order structural resolution of regulatory activity. The majority of the CRDs contained two REs, while some captured correlated activity among >80 REs (mean number of REs per CRD 4.7; mean CRD length 138 kb in the combined set; Supplementary Fig. 7). Eighty-six percent of the CRDs lied within DLPFC TADs [10] (Supplementary Fig. 9a) with CRDs regrouping the same pairs of peaks as TADs (Supplementary Fig. 9b) and 80% of the peaks clustered similarly into CRDs as detected in HBCC cohort samples in Girdhar et al. [22]. (Supplementary Fig. 10).

**Table 1:** Molecular phenotype associations at FDR 5% in SCZ cases and controls.

Molecular phenotype and type of association	Tested sample		
GENE EXPRESSION	SCZ (*n* = 83)	CTL (*n* = 160)	SCZ + CTL (*n* = 243)
Differentially expressed genes	1363 (937↑, 426↓)		
eQTL	987	6716	11,242
SCZ-specific eQTL	158		
CRD	SCZ (*n* = 74)	CTL (*n* = 119)	SCZ + CTL (*n* = 193)
cQTL	3239	13,158	28,538
CRDs	10,938	10,376	11,374
Differentially active CRDs	1141 (542↑, 599↓)		
aCRD-QTL	857	3144	6078
SCZ-specific aCRD-QTL	42		
QTL-CRD-GENE	SCZ (*n* = 59)	CTL (*n* = 105)	SCZ + CTL (*n* = 164)
Gene-CRD pairs	95/119*	634	1197
eCRDQTL-CRD-gene triplets	36	533	1134

The columns indicate the molecular phenotype and type of association, and the numbers of identified associations and sample set used.

*Gene-CRD associations found using CRDs identified in the combined set or CRDs identified only in SCZ cases.

**Fig. 1:**
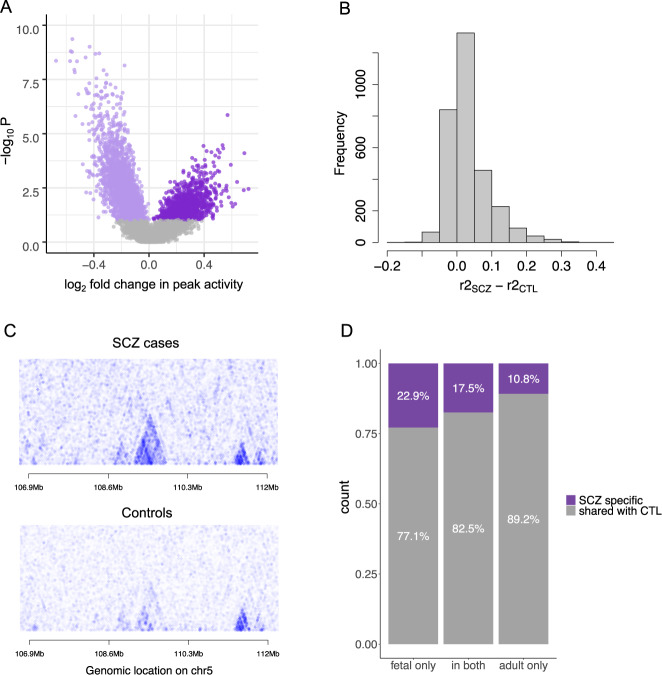
Features of SCZ-specific CRDs. **A** Difference in medians per peak activity between SCZ cases and controls as a function of the strength of association given in -log10 *p*-values; purple dots denote peaks that are differentially active between SCZ cases and controls at FDR 5% (3450 peaks) with light purple and dark purple indicating lower and higher median activity, respectively, in SCZ cases compared to controls. **B** Comparison of differences in per CRD peak activity correlation estimates between SCZ cases and controls for SCZ-specific CRDs (3078 CRDs). **C** Example region of a correlation structure between 250 peaks on chromosome 5 in SCZ cases and controls, revealing a well-delimited SCZ-specific CRD that is composed of five regulatory elements. The higher the correlation between peaks, the darker the colour blue. **D** Proportion of SCZ-specific CRD peaks that overlap with active regulatory regions identified in fetal samples (Fisher’s exact test *p* = 0.001, OR = 1.52) compared to those captured in adults (Fisher’s exact test *p* = 3.04 × 10^−50^, OR = 0.57).

Given that not all peaks regrouped into same CRDs in SCZ cases and controls (Supplementary Fig. 8a), we sought to investigate the mechanism for SCZ-specific CRD formation. Specifically, we asked whether the chromatin peaks within SCZ-specific CRDs were differentially active or whether these had larger variance in activity in controls compared to SCZ cases. To this end, we considered CRDs in SCZ cases composed of peaks not part of any CRD in controls (3078 CRDs composed of 6650 peaks) and compared single peak activities and mean correlation estimates among peaks per CRD between the two groups. We discovered that 53% of the peaks within SCZ-specific CRDs (3,540 peaks in 2,212 CRDs) were differentially active in SCZ cases at FDR 5% (Supplementary Fig. 11a). While the majority of the peaks (71%) showed lower activity in SCZ cases (Fig. 1A), only a third of SCZ-specific CRDs had all underlying peaks differentially active between SCZ cases and controls (Supplementary Fig. 11b), implying that while the regulatory activity originating from those genomic regions likely results in inhibition of downstream molecular cascades, difference in activity is not the main mechanism for SCZ-specific CRD formation. The peaks of SCZ-specific CRDs displayed significantly higher mean correlation in SCZ cases compared to controls (Mann-Whitney U test *p* = 5.02 × 10^−47^; Fig. 1B, C), indicating that changes in the 3D structure of the genome, rather than differential activity, were responsible for SCZ-specific CRD formation.

The peaks within SCZ-specific CRDs were significantly enriched for DLPFC H3K27ac peaks detected in fetal samples only [38] (Fisher’s exact test *p* = 0.001, odds ratio (OR) 1.52) and significantly depleted of those captured in adults [38] (Fisher’s exact test *p* = 3.04 × 10^−50^, OR = 0.57; Fig. 1D, Supplementary Table 3). Furthermore, compared to peaks that regrouped into CRDs in both SCZ cases and controls, the SCZ-specific CRD peaks were significantly enriched for SCZ GWAS variants (*p* = 0.008; Supplementary Fig. 12, Supplementary Table 4). At FDR 5%, eleven SCZ-specific CRDs were associated with the expression of proximal genes, for example *POU3F1* (also known as *OCT6*, transcriptional repressor for myelin-specific genes [39]), *KIF5A* (neuronal-specific vesicular transporter [40]), *NECAB1* (Ca^2+^-binding in neurons [41]), and *PDCD1LG2* (immune checkpoint receptor ligand [42]) (Supplementary Table 5). These associations exemplify coordinated regulatory changes specific to the disease state that affect or are affected by gene expression perturbations.

### Changes in CRD activity track alternations in gene expression in SCZ

**Fig. 2:**
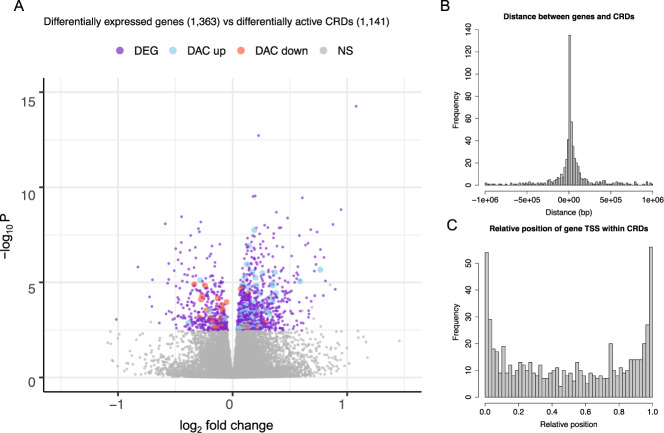
Association between CRDs and genes. **A** Expression of differentially expressed genes (DEGs) is correlated with the activity of differentially active CRDs (DACs) significantly more often than expected by chance (Fischer’s exact test *p* = 3.43 × 10^−5^, OR = 1.65); coloured dots denote DEGs genome-wide identified at FDR 5%: purple dots mark DEGs, blue dots mark DEGs whose expression correlates with the activity of a DAC in the same direction, red dots mark DEGs whose expression correlates with the activity of a DAC in the opposite direction. **B** Distribution of gene-to-CRD distances for genes localizing outside the associated CRD boundary (545 gene-CRD associations). **C** Distribution of the relative position of gene TSS to the boundary of an associated CRD (652 gene-CRD associations).

Having identified several distinct RE coordination structures in DLPFC in SCZ cases that were absent in controls, we focused next on CRDs that had the same structure of RE coordination across SCZ cases and controls (i.e., CRDs identified in the combined set). We set out to determine the differences in regulatory activity between SCZ cases and controls and to investigate their relation to genes that were differentially expressed (DEGs) between the two groups. At FDR 5%, we identified 1141 CRDs (599 lower activity, 542 higher activity) and 1363 genes (937 up-regulated, 426 down-regulated) to be differentially active and expressed in SCZ cases, respectively (Table 1, Supplementary Fig. 13a, b, Supplementary Table 6, Supplementary Table 7). The differences in effect size for CRDs were subtle, reflecting a narrow variability range in regulatory activity (Supplementary Fig. 13a). The determined DEGs were in concordance with those previously reported in SCZ pathogenesis (Supplementary Fig. 14) [9, 43] and were significantly enriched for gene ontology (GO) terms related to sex-hormone and interferon-*γ*-mediated signalling, glucocorticoid receptor and glutamate receptor binding, axonogenesis and synapse assembly (Supplementary Table 8). Up-regulated DACs were significantly enriched for SCZ GWAS variants (*p* = 0.002), whereas down-regulated DACs were not (Supplementary Fig. 12, Supplementary Table 4). Moreover, DEGs were significantly enriched for differentially active CRDs either based on nominal correlation (91 DEGs correlated with DACs; Fisher’s exact *p* = 3.43 × 10^−5^, OR = 1.65) (Fig. 2A), or based on genomic location (transcription start site (TSS) of 118 DEGs lied within differentially active CRDs; Fisher’s exact test *p* = 8.62 × 10^−6^, OR = 1.60; Supplementary Fig. 13c). The majority of DEGs (80%) showed the same effect direction as the CRD whose activity correlated with the gene’s expression or as the CRD in which the gene TSS lied (Supplementary Table 7), indicating that deviations in gene expression track alterations in the regulatory machinery.

Correlating gene expression and CRD activity (i.e., CRDs identified in the combined set) in cis using linear regression revealed 95, 634, and 1197 CRD-gene associations in SCZ cases, in controls, and in the combined set, respectively, at FDR 5% (Table 1, Supplementary Fig. 15, Supplementary Table 9). The majority of the genes were associated with a single CRD and the majority of the CRDs with a single gene with only a handful of CRDs being associated with up to ten different genes (Supplementary Fig. 16). Most gene TSSs clustered at CRD boundaries (Fig. 2B, C), corroborating the proximal role of coordinated regulatory activity in gene transcription.

### Genetic regulation of CRD activity and gene expression in SCZ

**Fig. 3:**
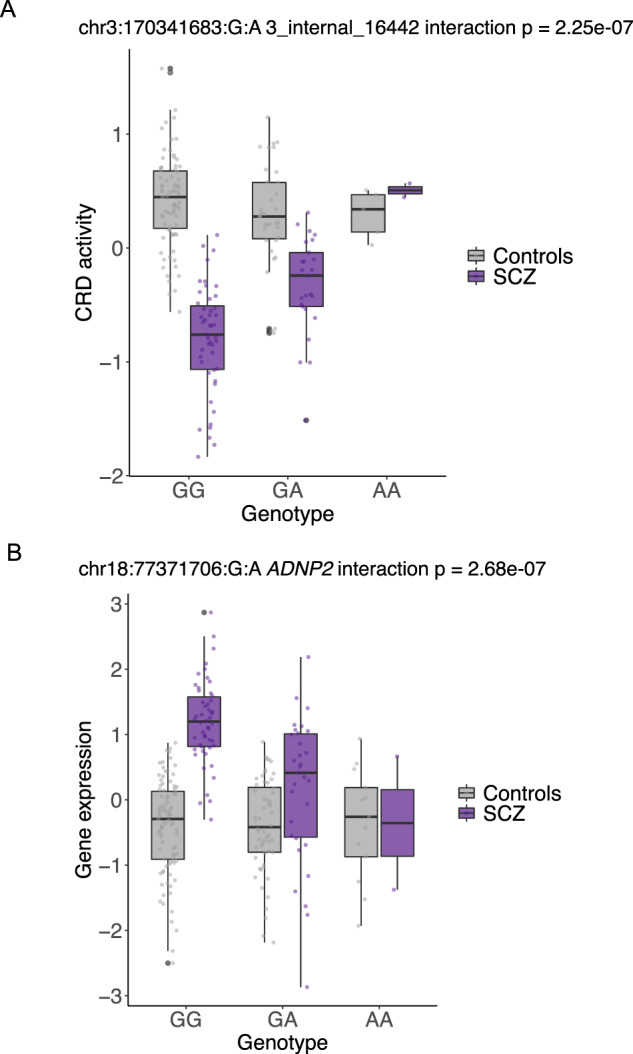
SCZ-specific QTL effects. Genotype-dependent effect on (**A**) CRD activity and (**B**) gene expression identified only in SCZ cases.

We next sought to study the genetic regulation of CRD activity and gene expression, search for SCZ-specific QTL effects and interrogate whether QTL effects colocalize with SCZ risk variants. At 5% FDR and in cis, we discovered 857 and 3,144 functionally independent CRD activity QTLs (aCRD-QTLs), and 987 and 6,716 functionally independent eQTLs in SCZ cases and controls, respectively (Table 1, Supplementary Table 10, Supplementary Table 11, Supplementary Fig. 17). The strength of the association was correlated with the genomic distance from the molecular phenotype (Supplementary Fig. 18). While almost all SCZ-identified QTL effects replicated in controls (Supplementary Fig. 19), 5% of aCRD-QTLs (*n* = 42) and 16% of eQTLs (*n* = 158) showed SCZ-specificity, i.e., these affected CRD activity or gene expression only in SCZ cases or displayed significant change in effect size compared to controls (Table 1, Fig. 3A, B, Supplementary Table 10, Supplementary Table 11). The SCZ-specific genotype-dependent variability in CRD activity and in gene expression imply context-dependent and pathway-activated gain in regulatory capacity. Results of gene enrichment analysis for genes with SCZ-specific eQTLs conform with posed hypotheses linking dysregulation of glutathione binding and adenosine deaminase activity with SCZ [44–46] (Supplementary Table 12). Colocalization analyses for SCZ risk variants with aCRD-QTLs and with eQTLs showed modest yet proportionally similar enrichment for shared functional effects (1.3% for SCZ-identified aCRD-QTLs and 1.6% for SCZ-identified eQTLs). Interestingly, the aCRD-QTLs colocalized with different GWAS variants compared to eQTLs that shared a functional effect with SCZ risk variants (Supplementary Table 13). Of H3K27ac peaks with a QTL signal detected in the PsychENCODE resource [10], an estimated 56% (π1 statistic [28]) showed a QTL effect for a peak that regrouped into a CRD in the current study (Supplementary Fig. 20).

To assess common genetic regulation of coordinated regulatory activity and gene expression, we investigated the association of aCRD-QTL and eQTL effects on the other molecular phenotype. Specifically, we correlated aCRD-QTLs with gene expression and eQTLs with CRD activity over gene-CRD associations detected across all samples at nominal significance. We identified that up to 47% of the eQTL and aCRD-QTL variants had an effect on CRD activity and gene expression, respectively (Supplementary Fig. 21). The considerable overlap between aCRD-QTL and eQTL effects for relevant CRD-gene pairs corroborates the functional interplay among genetic variants, CRDs, and genes.

### Refining eQTL perturbations reveals regulatory machinery changes specific to SCZ

Given the established interplay among genetic variants, genes, and CRDs, we interrogated the functional directionality between them. We reasoned that the effect of a genetic variant on gene expression could either be mediated by or propagated to the changes in coordinated RE activity and that deviations in the regulatory machinery in SCZ cases compared to controls would imply molecular dysregulation specific to disease. To test this, we considered the previously discovered 1197 CRD-gene pairs ascertained across SCZ cases and controls at FDR 5% and identified the same genetic variant (eCRD-QTL) that affected both molecular phenotypes and by that determined eCRDQTL-CRD-gene triplets for causal inference. Using Bayesian Networks (BN), we tested three relationship patterns: (i) causal model in which the genetic variant affects first the activity of the CRD which then regulates the gene expression, (ii) reactive model in which the genetic variant affects the expression of the gene which then modulates the CRD activity, and (iii) independent model in which the genetic variant affects the gene and the CRD independently; and studied these relationships separately in SCZ cases (*n* = 59) and controls (*n* = 105) (Supplementary Fig. 22). We discovered that at FDR 5%, 95% of the CRD-gene pairs had a cis-QTL effect (*n* = 1134; Table 1), indicating that the simultaneous change in CRD activity and gene expression was affected by the same nearby genetic variant. We observed more causal models in controls than in SCZ cases (Supplementary Fig. 23a, b), which were likely driven by the smaller SCZ sample size as reflected by the distribution of the probabilities for the most likely model for each triplet (Supplementary Fig. 23c, d). The probability of the causal model increased the further the gene TSS was from the eCRD-QTL in both SCZ cases and controls (Supplementary Fig. 23e, f), denoting the role of CRDs mediating the genetic effect onto distal genes.

**Fig. 4:**
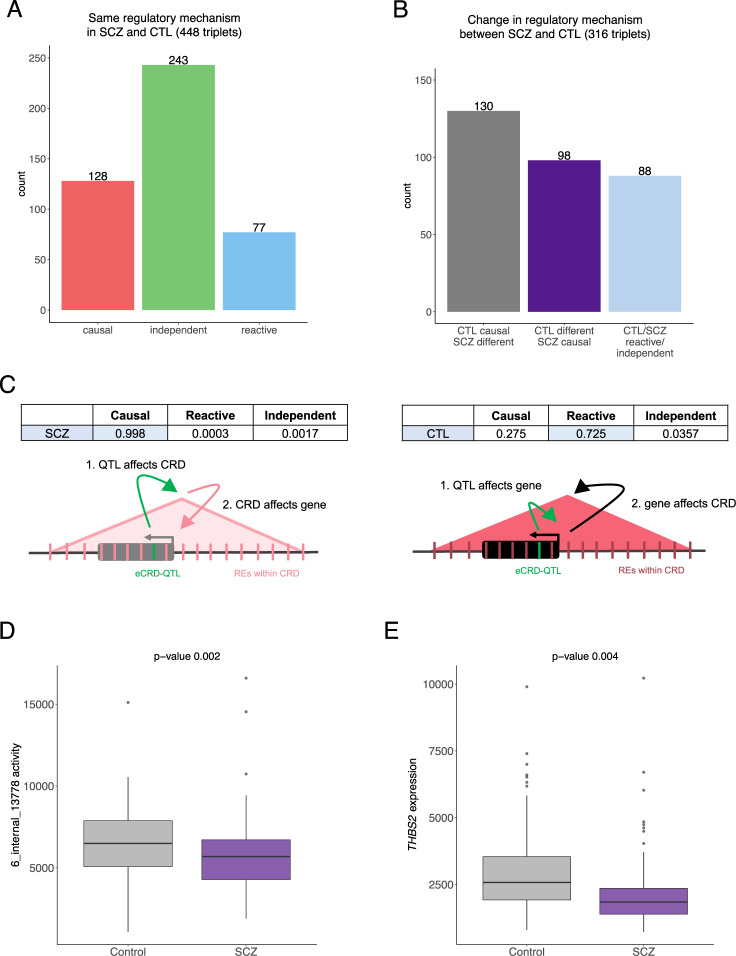
Regulatory mechanisms for eCRDQTL-CRD-gene triplets in SCZ cases and controls. Comparison of the direction of effect from eCRD-QTL onto gene expression and CRD activity for tested triplets between SCZ cases and controls: **A** triplet count for models showing the same regulatory mechanism in SCZ cases and controls, and **B** triplet count for models showing a change in the regulatory mechanism between SCZ cases and controls (term “different” indicates either reactive or independent model; light blue bar indicates triplets, for which the causal model (i.e., mediation via CRD for QTL effect) was not identified in SCZ cases nor in controls). **C** Distinct regulatory mechanism of the genetic regulation on gene expression for SCZ cases and controls for a triplet consisting of an eCRD-QTL chr6:169646282:A:T, gene *THBS2* and a CRD composed of 18 REs on chr6:169,541,739–169,999,929; the probabilities based on the BN analysis for each tested model is given above schematics; shading of the colour for the gene and for the CRD indicates strength in expression and activity, respectively, relative to the other disease status group (SCZ cases vs controls). **D**, **E** Distribution of CRD activity and *THBS2* gene expression for SCZ cases and controls.

To study the proportion of differentially regulated mechanisms between SCZ cases and controls and ascertain which molecular functions were affected by these changes, we first estimated the accuracy for BN results using bootstrapping to provide confidence for retrieved probabilities and next carried out gene enrichment analysis for genes associated with different regulatory mechanisms. The accuracy for inferring the most likely causal relationship for triplets was lower in SCZ cases (mean accuracy estimation 68.3%, sd = 16.8) than in controls (mean accuracy estimation 75.1%, sd = 17.6) (Supplementary Fig. 24a, b). To exclude ambiguous signals, we proceeded with triplets that surpassed the accuracy estimation of 55% (764 triplets, 67.3% of studied triplets; Supplementary Fig. 24c, Supplementary Table 14). While two-thirds of the triplets displayed the same regulatory mechanism in SCZ cases and controls (Fig. 4A), one-third of studied triplets showed a change in the directional effect from QTL variant onto molecular phenotype (Fig. 4B, Supplementary Fig. 24d). These deviations in regulatory mechanism in SCZ reflect gain or loss in the regulatory capacity that could either be driven by context-dependent or genetically predisposed developmental derailment of gene expression, or affected by external stimuli (e.g., treatment). The genes associated with change-associated triplets were enriched for GO terms related to small GTPase binding, and filopodium assembly (Supplementary Table 15), highlighting alterations in the regulatory machinery for gene expression affecting synaptic function and plasticity, and dendritic spine morphology in SCZ [47–49].

A perturbation in the regulatory mechanism of gene expression in SCZ is exemplified by a triplet consisting of an eCRD-QTL chr6:169646282:A:T associated to gene *THBS2* and to a CRD composed of 18 REs (chr6:169,541,739–169,999,929). THBS2 is an extracellular matrix protein of the central nervous system that is secreted by astrocytes to control excitatory synaptogenesis. Importantly, small GTPase proteins have been shown to play a key component in the synaptogenic signalling cascade downstream of the protein and its neuronal calcium channel subunit receptor [50–52]. Based on BN, the genetic variant affected first the activity of the CRD and then the expression of the gene in SCZ cases (causal model probability 0.99), whereas in controls the change in CRD activity was a reaction to gene expression (reactive model probability 0.72) (Fig. 4C). While the eCRD-QTL displayed the same effect direction on gene expression and on CRD activity in SCZ cases and controls (Supplementary Fig. 25a, b), both the CRD and the gene were significantly downregulated in SCZ samples compared to controls (p-value 0.002 and 0.004, respectively; Fig. 4D, E), indicating that the eCRD-QTL effect on *THBS2* expression did not translate via the same regulatory mechanism in both states. Interestingly, this association was identified only in individuals with African American ancestry (Supplementary Fig. 25c, d) as the genetic variant was completely monomorphic in HBCC Europeans. The MAF spectrum of 5% in HBCC African Americans and 0% in HBCC Europeans is in concordance with population frequencies estimated in larger datasets (MAF 5% in Africans/African Americans and 0.0002% in non-Finnish Europeans) [53]. These results indicate that the downregulation of *THBS2* expression in SCZ cases was mediated by the regrouping of the identified regulatory regions and represents a dysregulated step within an abnormal molecular cascade affecting synapse function in SCZ.

## Discussion

Deciphering regulatory mechanisms of gene expression that reflect molecular perturbation in SCZ are under extensive scrutiny yet are hindered by the complexity of the SCZ phenotype and scarcity of relevant molecular data. Studying regulatory activity that tracks changes in gene expression requires a higher order analysis approach for signal discernment due to narrow variability range in regulatory activity and extensive multiple testing burden [12, 22]. Here we show that taking account of interindividual correlation between regulatory activity allows to refine changes in gene expression specific to disease, asserting that disease manifestation stems from dysregulated gene expression cascades that are steered by and propagated to the concerted action of REs. Interrogation of common genetic regulation of gene expression and CRD activity corroborated that correlated changes in gene expression and CRD activity are affected by the same genetic driver. Our results agree with findings showing considerable overlap between QTL effects on chromatin accessibility and gene expression [12], that a single genetic variant drives the association between multiple chromatin peaks and a single gene [20], and on the convergence of deviations detected in different molecular layers as seen for gene expression and methylation [54] and for gene expression and acetylated histone peaks [22]. Applying causal inference to study the causal relationships among genetic variants, genes and CRDs revealed regulatory machinery changes affecting synaptic function and dendritic spine morphology in SCZ which are in line with established molecular abnormalities identified for the disorder [47–49]. The deviations in regulatory mechanisms reflect gain or loss in the regulatory capacity that could either stem from genetic predisposition, are acquired in disease progression or result from chronic pharmacology. Clear discernment of the proposed origins of effect was hampered due to small sample size and unavailability of relevant data yet allowed to draw the following conclusions.

First, our results support the neurodevelopmental hypothesis for schizophrenia. Multiple studies have highlighted the concordance of SCZ heritability enrichment for open chromatin regions in fetal and in SCZ DLPFC samples and the stability of methylation and expression feature deviations in fetal brain development that persist into adulthood for those affected by the disorder [9, 12, 13, 22, 55]. Using DLPFC H3K27ac peaks captured within the PsychENCODE Human Brain Developmental resource [38], we identified significant enrichment of SCZ-specific CRDs for fetal vs adult regulatory activity. Furthermore, peaks either within SCZ-specific CRDs or within up-regulated DACs showed significant enrichment for SCZ GWAS variants, replicating the findings by Girdhar et al. [22], and implying that a considerable proportion of signals detected in the current analysis reflect brain development derailment due to genetic predisposition for SCZ. While we found modest colocalization for detected QTLs with SCZ-predisposing genetic variants, the results reflect direct correlation between sample size and QTL signal detection [56] and hence are in proportion to colocalization signals ascertained in previous findings [9, 12, 43, 57]. Second, while molecular data used in this study was extracted from bulk tissue, consistent comparison with signals identified in control samples provided confidence that the identified deviations in SCZ cases captured the most notable disease-specific molecular abnormalities in DLPFC. This is further supported by investigations revealing that DLPFC transcriptomic profiles are generally biased toward neuronal populations [54], that SCZ risk variants are overrepresented in neuronal vs non-neuronal open chromatin regions [58], and that SCZ-specific deviations detected for CRDs identified in DLPFC neuronal NeuN^+^ nuclei samples overlap with those detected in bulk DLPFC [22]. Third, studying QTL effects on gene expression and on CRD activity separately in SCZ cases and controls allowed to discriminate context-dependent genetic effects on both molecular phenotypes, indicating gain in regulatory capacity that translated into gene expression and coordinated regulatory activity variability only in SCZ cases or showed significantly different effect between the two groups. Fourth, while the unavailability of treatment information for the HBCC cohort precluded testing whether molecular-level differences in SCZ cases were impacted by pharmacological effects, previous studies that showed concordant DEG signals **(**Supplementary Fig. 14b) and H3K27ac peak content (Supplementary Methods) with those found in the current analysis, identified that differential gene expression and chromatin alterations in SCZ were not driven by antipsychotic intervention [22, 43], suggesting that treatment effect could not have been the main trigger for differential analyses results between SCZ cases and controls. Fifth, while we applied a PC analysis-based approach to capture any biological and technical variability in molecular phenotype quantifications, our results of BN analysis using gene expression and CRD quantification data could have been affected by unmeasured and uncaptured confounders, given that we see larger error in detecting cQTLs compared to eQTLs (Supplementary Fig. 26). Lastly, inclusion of individuals of European and African American ancestry augmented signal identification and corroborates that the genetic basis of SCZ and its biology are broadly shared across populations [7, 34, 59].

Altogether, we have outlined that leveraging higher-order structural resolution of regulatory activity allows to reduce the search space for unveiling genetically perturbed regulation of gene expression specific to SCZ. We anticipate that cell-type specific gene expression and open chromatin exposure profiles in larger sample sets would allow better delimitation of CRD chromatin peak content, facilitate the identification of trans-regulatory hubs across different chromosomes as well as enhance more robust detection of origin effect for gene expression deviations, thereby increasing our understanding of perturbed functional pathways underlying schizophrenia and for prioritizing targets for experimental investigation and novel treatment development.

## Supplementary information

Supplementary Information

Supplementary tables

## Data Availability

Phenotype and molecular data for the HBCC cohort are deposited at dbGaP (study accession phs000979.v3.p2). Genotype data for 1000 Genomes Project are deposited at https://www.internationalgenome.org/data-portal/data-collection.
