# Supplementary material for: Leveraging interindividual variability of regulatory activity for refining genetic regulation of gene expression in schizophrenia
Source: Mol Psychiatry. 2022 Sep 16;27(12):5177–85. doi: 10.1038/s41380-022-01768-4 (PMC9763101; doi:10.1038/s41380-022-01768-4)
Supplement: Supplementary file 1 — Supplementary Information [file 41380_2022_1768_MOESM1_ESM.pdf]

Supplementary Information

Leveraging interindividual variability of regulatory activity for refining genetic regulation of gene expression in schizophrenia

Maris Alver[1,2,3,4]*, Nikolaos Lykoskoufis[1,2,3], Anna Ramisch[1,2,3],

Emmanouil T. Dermitzakis[1,2,3] & Halit Ongen[1,2,3]*

[1] Department of Genetic Medicine and Development, University of Geneva, Geneva, Switzerland
[2] Swiss Institute of Bioinformatics, University of Geneva, Geneva, Switzerland
[3] Institute of Genetics and Genomics in Geneva, University of Geneva, Geneva, Switzerland
[4] Estonian Genome Center, Institute of Genomics, University of Tartu, Tartu, Estonia

This PDF file includes:
Supplementary Materials and Methods
References for Supplementary Materials and Methods
Supplementary Figures S1 to S26

DATA PREPARATION

General overview

Molecular data for the Human Brain Collection Core (HBCC) within the Division of Intramural Research Programs (DIRP) at the National Institute of Mental Health (NIMH) was accessed through dbGaP (study accession phs000979.v3.p2; request #88083-1 approved by NIH on January 31st, 2020; https://www.ncbi.nlm.nih.gov/projects/gap/cgi-bin/study.cgi?study_id=phs000979.v3.p2).

Briefly, post-mortem dorsolateral prefrontal cortex (DLPFC) brain tissues were obtained under the protocols approved by the CNS IRB with the permission of the next-of-kin through the Offices of the Chief Medical Examiners in the District of Columbia, Northern Virginia, and Central Virginia, and from the University of Maryland Brain and Tissue Bank and the Stanley Medical Research Institute. Clinical characterization, neuropathological screening, toxicological analyses, and the dissections of the brain region were performed as described previously(1). All patients met DSM-IV criteria for a lifetime Axis I diagnosis of psychiatric disorders including schizophrenia or schizoaffective disorder. Controls had no history of psychiatric diagnoses or addictions. While the HBCC cohort consists of individuals with different ancestral background, we opted for using samples from the two largest self-reported ancestral groups: African American and European.

Phenotype data

At least two levels of molecular data (i.e., whole-genome genotype, RNA-sequencing or ChIP-sequencing data) were available for 272 individuals: 98 SCZ cases and 174 controls, 188 males and 84 females, 164 African Americans and 108 Europeans; controls <16 years of age were excluded (**Supplementary Fig. 2**).

Genotype data

Subjects were genotyped with three Illumina whole-genome genotyping arrays: HumanHap650Y, Human1M-Duov3 and HumanOmni5M-Quad. Raw .idat files were downloaded from dbGaP and converted to .gtc and .vcf files using Illumina Array Analysis Platform Genotyping Command Line Interface (iaap-cli and gtc2vcf, respectively; webpage: https://github.com/freeseek/gtc2vcf). Array-based quality control was carried out with PLINK v2.0(2) (webpage: https://www.cog-genomics.org/plink/2.0/) using the following criteria: i) exclusion of individuals with genotype call rate <95%; and exclusion of single nucleotide variants (SNVs) with call rate <95%, Hardy-Weinberg equation (HWE) <10e-5, minor allele frequency (MAF) <0.01, and with ambiguous genotypes (AT and GC SNVs); ii) confirmation of the match for genotype and phenotype sex, removal of outliers who deviated +/- 3 standard deviations from the samples' heterozygosity rate mean as well as verification that the data did not contain closely related individuals (PI_HAT >0.2).

Prior to imputation, we determined the intersect of SNVs across three genotyping arrays, filtered each array for the uniform content (506,548 SNVs) and merged the data into one matrix. We used the TOPMed Imputation Reference panel(3) (webpage: https://imputation.biodatacatalyst.nhlbi.nih.gov/) for array imputation with the following parameters: build hg19, reference panel apps@topmed-r2@1.0.0 (GRCh/hg38), population all, phasing eagle. After imputation, we filtered out SNVs with low imputation quality score R2 <0.3 and applied HWE <1e-6 and MAF <0.05 filters by European and African American ancestral group separately. For the final SNV set, we considered the union of filtered SNVs retrieved in both ancestry sets (9,516,522 SNVs). Given that the TOPMed Imputation Server outputs SNVs on GRCh/hg38, Picard LiftoverVcf tool (webpage: https://broadinstitute.github.io/picard) was used to lift SNVs back to hg19 based on the UCSC chain file (hg38ToHg19). We tested for an association between imputed SNVs and genotyping arrays to confirm that SNV data were not biased towards any genotyping array. No bias towards any genotyping array was detected (**Supplementary Fig. 3**).

We used the 1000 Genome Project data(4) as reference to exclude samples that showed differential ancestry background than European or African/Admixed American based on the principal component analysis (PCA) (**Supplementary Fig. 4**).

RNA-sequencing data

Raw .fastq files (2 x 125bp pair-end reads) were downloaded from dbGaP. RNA-seq mapping was done with STAR v2.7 2-pass mapping(5) using GENCODE hg19 reference genome annotations. RNA-seq data quality was assessed using QTLtools bamstat mode(6) (webpage: https://qtltools.github.io/qtltools/) for i) the number of mapped sequencing reads passing mapping quality filter, ii) number of mapped sequencing reads falling within the GENCODE hg19 annotations, and iii) the number of GENCODE hg19 annotations covered by at least one sequencing read.

Gene expression was quantified by counting the number of RNA-seq reads that were in correct orientation, that had a mapping quality score above 225 and that did not contain more than 16 mismatches in both ends of the fragment with the reference genome. Each gene in each sample was normalized to get an RPKM value using the formula RPKM = (read_count x 1e9) / (total_mapped_reads x gene_length). We filtered for protein-coding and lincRNA genes and considered the union quantifications detected in >=50% in SCZ cases and in >=50% in controls. This yielded 18,258 protein-coding genes and 3,730 lincRNAs (total of 21,988 genes) for 243 individuals.

ChIP-sequencing data

Raw .fastq files (2 x 75bp pair-end reads; profiled for histone mark H3K27ac) were downloaded from dbGaP. Reads were aligned to reference genome hg19 with BWA-MEM v0.7.16(7) using an alignment score threshold of 10. For quality control, all ChIP-seq experiments were processed through Phantompeakqualtools v1.16(8) to generate two quality metrics: normalized strand cross-correlation (NSC) and relative strand cross-correlation (RSC) (**Supplementary Fig. 5**). These metrics use the cross-correlation of stranded read density profiles to measure enrichment independently of peak calling. RSC <0.8 and NSC <1.05 indicate low signal to noise ratio. The quality tag (based on thresholded RSC) was >2 (very high) for all experiments.

Peak calling and quantification were carried out with HOMER v14.11.1(9) (webpage: http://homer.ucsd.edu/homer/). Briefly, we first determined ChIP-seq peak coordinates across SCZ cases and controls to get a population scale call set of ChIP-seq peaks and then quantified the peaks for each individual according to the identified peak coordinates. Specifically, we first built a population call set of ChIP-seq peaks by aggregating 1e6 ChIP-seq reads from 74 SCZ cases and 74 controls together in a unique BAM file. Next, we carried out the actual peak calling onto the derived consensus peak set (derived a unique BAM file) for all individuals (n=193: 74 SCZ cases and 119 controls) using HOMER findPeaks mode and parameters -style histone -o auto. This yielded 141,218 ChIP-seq peaks for each individual (mean peak length 2,137 base pairs). Next, we quantified the peaks by obtaining per-peak read counts per sample using the peak coordinates from the consensus peak set and using the HOMER script annotatePeaks.pl with the following options: - noann -nogene -size given. This script counted the number of ChIP-seq reads falling within the peak coordinates. Read counts were subsequently normalized for a total of 10 million mapped reads per sample.

Mislabelling detection

Given that many analyses relied on testing genotype versus sequence data, we looked at the concordance between both to ensure there was no sample mislabelling and all individuals had both sequence and genotype data available. We used QTLtools mbv mode(6, 10) and .vcf and .bam file for each individual and assessed the concordance at heterozygous and homozygous genotypes between genotype and RNA-sequencing and between genotype and ChIP-seq sequencing data. No such errors were detected.

COMPUTATIONAL AND STATISTICAL ANALYSES

Cis-regulatory domain (CRD) calling

For CRD calling, we used the pipeline developed in Delaneau et al. 2019(11) (webpage: https://github.com/odelaneau/clomics). We started with building a correlation map by systematically measuring interindividual correlation between all possible pairs of ChIP-seq peak quantifications (i.e., retrieved Pearson correlation coefficients using corrected and rank-normal transformed data matrix (see final paragraph of this section for specifics). Next, we applied an agglomerative hierarchical clustering of the data on a per chromosome basis. Specifically, we started with each ChIP-seq peak being assigned to its own cluster and iteratively merged clusters as we moved up in the hierarchy. To determine the pair of clusters to be merged, we maintained throughout the procedure a correlation matrix that corresponded to the matrix of squared correlations between ChIP-seq peaks, searched the pair of clusters that exhibited the highest value as well as constantly updated the correlation matrix when merging clusters together. For minimizing computational cost, we searched for the maximal correlation between clusters that were not separated by more than 250 rows or columns (i.e., peaks or groups of peaks in the correlation matrix), allowing to store and update the diagonal parts of the correlation matrix and merging together proximal features of the genome. This strategy resulted in a binary tree that regrouped all ChIP-seq peaks from the same chromosome in which each node delimited a set of highly correlated ChIP-seq peaks.

CRDs were called by identifying the minimal set of internal nodes that captured most of the overall correlation mass (i.e., cumulative sum of squared correlation). To retain an internal node as a CRD, three criteria needed to be fulfilled: i) CRDs regrouped only highly correlated ChIP-seq peaks: the mean absolute correlation between all possible pairs of ChIP-seq peaks within a CRD had to be at least twice as high as the mean correlation between all ChIP-seq peaks in the chromosome; ii) CRDs had well-defined boundaries: the mean absolute correlation between all pairs of ChIP-seq peaks involving either the first or the last ChIP-seq peak (on the basis of their genomic location) had to be at least twice as high as the same value derived for the first and last peaks on the chromosome; iii) CRDs captured distal coordination between at least two regulatory elements (REs): ChIP-seq peaks had to cover at least two non-overlapping regulatory regions. These three criteria were implemented into an algorithm that processed each binary tree starting from the root node (node regrouping all peaks of a chromosome) and recursively traversed the internal nodes of the tree until an internal node fulfilled all three criteria; then, declared the internal node and all the peaks downstream as a CRD, stopped to go deeper by ignoring the children of this node and carried on with other internal nodes in the tree.

This pipeline was applied using only SCZ ChIP-seq quantifications (n=74), only control ChIP-seq quantifications (n=119) and using ChIP-seq quantifications in the combined set (i.e., SCZ cases and controls together; n=193), such that we started with three separate correlation maps and in the end had called SCZ-identified CRDs, controls-identified CRDs as well as uniform CRDs across all samples. For correcting ChIP-seq quantifications, we identified the optimal number of PCs that captured variability in ChIP-seq data in SCZ cases, in controls and across samples via QTL mapping (see section QTL mapping for molecular phenotypes) by considering variable number of PCs as covariates. After finding the optimal configuration (giving the best QTL discovery power; **Supplementary Fig. 6a-c**), we corrected ChIP-seq peak quantifications for 3 genotype PCs and 10 ChIP-seq PCs in SCZ cases (n=74), 3 genotype PCs and 20 ChIP-seq PCs in controls (n=119), and 3 genotype PCs and 30 ChIP-seq PCs across samples (n=193). All three resulting data matrices were rank-normal transformed separately.

We additionally carried out CRD calling in controls by down-sampling to the same number of individuals as SCZ cases, i.e., n=74. This resulted in the identification of 187 additional CRDs, yet contained less peaks in total: 10,563 CRDs in down-sampled set grouping together 40,156 peaks in total vs 10,376 CRDs in 119 controls grouping together 44,391 peaks in total. A larger sample size allows us to discover more CRDs that capture correlated activity across multiple regulatory regions (mean 4.3), whereas using a smaller sample set resulted in higher number of CRDs with lower number of correlated peaks (mean 3.8) (**Supplementary Fig. 7bd**). An overlap analysis between CRD peak content identified in 74 SCZ cases and in down-sampled controls revealed fewer CRDs (35%) to be shared between the two states compared to the original analysis where we used 74 SCZ cases and 119 control samples and identified 42% of the CRDs to be

shared (**Supplementary Fig. 8**). For determining CRD sharing between SCZ cases and controls, we compared ChIP-seq peak correlation maps between SCZ cases and controls and called a CRD shared if ≥50% of the peaks overlapped between the reference and the query correlation map. This analysis allowed us to deduce that using a larger sample set in controls provides power to capture more peaks that show correlation and group into CRDs, thus minimizing the concern of cryptic SCZ-specific CRD discovery using an unbalanced sample set.

<u>CRDs in relation to topologically associated domains (TADs)</u>

To ascertain the relation of CRDs to TADs, we considered TADs (n=2,375) identified in DLPFC samples within the PsychENCODE resource(12) and CRDs identified across SCZ cases and controls (n=11,374). A TAD and a CRD were determined to overlap in case the start and end of a CRD lied within TAD boundaries (BEDTools v2.28.0(13)). To ascertain whether peaks that regrouped into the same CRD also lied within the same TAD, we determined odds ratios from contingency tables for peak pairs at different distance bins (**Supplementary Fig. 9b**).

<u>Overlap between CRD peak coordinates with previous findings</u>

Given that CRDs calling was applied on the HBCC cohort samples using a different adjacency-constrained hierarchical clustering methodology in Girdhar et al.(14), we determined the overlap between the CRD peak coordinates in both studies. To this end, we considered the peak coordinates outlined in Girdhar et al., lifted these over to hg19 assembly using the UCSC hg38ToHg19 chain file and used BEDtools v2.28.0(13), requiring that 90% of the genomic content of the peaks detected in the current study overlapped with those detected in Girdhar et al. We identified that 92.3% of the peaks (130,455 peaks of all 141,128 considered) overlapped with the peak coordinates detected in Girdhar et al. and of the overlapping peaks that clustered into CRDs in both studies, 80% clustered into CRDs in our data (43,501 peaks in 8,965 CRDs of all 54,278 peaks in 11,374 CRDs considered). For H3K27ac peak calling, 133 controls, 68 SCZ cases and 48 bipolar disorder cases from HBCC were considered in Girdhar et al., and 119 controls and 74 SCZ cases from HBCC were considered in the current study.

<u>CRD activity quantification</u>

For CRD activity quantification, we applied a dimensionality reduction approach, i.e., we enumerated all ChIP-seq peaks per CRD, and took the mean of all single peak quantifications per individual to retrieve a single quantification value for each individual. We used the ChiP-seq peak correlation map retrieved in the combined set (i.e., across SCZ cases and controls; n=11,374 CRDs). For SCZ-specific CRD structure analyses, we retrieved CRD activity quantifications for SCZ cases only using the CRDs identified in SCZ cases (n=10,938 CRDs). The resulting vectors were rank-normalized such that these matched a normal distribution with mean 0 and standard deviation 1 and consisted of one row per CRD and one column per individual.

<u>Quantitative Trait Loci (QTL) mapping for molecular phenotypes (ChIP-seq peak activity, gene expression, CRD activity)</u>

For each molecular phenotype, we first enumerated all genetic variants within +/- 1 Mb and then tested each one of these variants for association with the phenotype and only retained the best hit (i.e., with the smallest nominal p-value). Secondly, we adjusted the best nominal p-value for the number of variants being tested by permutations. Specifically, we randomly shuffled the phenotype quantifications 1,000 times and retained the best association p-values for each permuted data set, which effectively gave 1,000 null p-values of associations. Third, to correct for the number of molecular phenotypes being tested whole genome (e.g., number of genes, peaks, CRDs), we used a false discovery rate (FDR) correction approach and declared phenotype-variant pairs at FDR 5% threshold as significant. These steps were carried out with QTLtools cis mode(6).

To discover multiple QTLs with independent effects on a given molecular phenotype, we used the conditional analysis approach implemented in QTLtools(6). Briefly, this approach is based on a forward-

backward scan of the cis-window around the phenotypes to automatically learn the number of independent QTLs and to identify the most likely candidate variants, while controlling for a given FDR.

For SCZ-specific QTL discovery, we considered QTL effects identified in SCZ cases (857 aCRD-QTLs and 987 eQTLs) and for each variant-phenotype pair ran a linear regression including genotype, disease status (SCZ/CTL), and covariates, and tested for significance of a genotype * disease status interaction on molecular phenotype (gene expression or CRD activity). This was followed with FDR 5% correction for the number of QTLs tested.

To determine the correlation between aCRD-QTLs identified across SCZ cases and controls and previously published signals, we used the PsychENCODE resource(12), which outlines 2,279 unique chromatin-QTL signals determined across 218 autism, schizophrenia, and control DLPFC samples profiled for H3K27ac at FDR 5% (DER-09). Given that PsychENCODE peaks had a uniform length of 999 bp and CRD peaks had a mean of 2137 bp, peaks in both independent datasets were determined as shared in case the centre of the peak that had a QTL signal in PsychENCODE lied within the peak that belonged into a CRD. This strategy resulted in the same overlap yield as if requiring 50% of the PsychENCODE peak to overlap a CRD peak (using BEDTools v2.28.0(13)). Proportion of QTL signal sharing was estimated using $\pi 1$ estimate(15). The same approach was applied for replicating PsychENCODE cQTL signals for individual cQTLs detected across SCZ cases and controls.

CRD structure analysis

To assess the features of SCZ-specific CRDs, we considered only CRDs in SCZ cases composed of peaks not regrouping into any CRD in controls. These formed 28% of the CRDs identified in SCZ cases (3,078 CRDs composed of 6,650 peaks). For underlying peak activity estimation, we used ChIP-seq peak quantifications normalized for 10 million reads per sample, uncorrected for any covariates and applied a Mann-Whitney U test per peak activity between SCZ cases and controls. As differential peak activity analysis is carried out in trans, i.e., comparison of peak activity distributions across the whole genome, correcting for ChIP-seq PCs would remove global effects. Significant differences between SCZ cases and controls were determined at FDR 5% using R/*qvalue* package(15).

For confirming whether the peaks within SCZ-specific CRDs showed different correlation structures in SCZ cases vs controls and were not driven by the mean background correlation estimate ascertained separately in SCZ cases and controls in CRD calling, we used ChIP-seq peak quantifications corrected for biological and technical covariates (3 genotype PCs and 10 ChIP-seq PCs in SCZ cases, and 3 genotype PCs and 20 ChIP-seq PCs in controls as outlined in CRD calling section). The corrected data matrices were rank-normal transformed separately. We calculated the mean Pearson correlation estimate between peak activities per CRD separately in SCZ cases and controls (i.e., in controls measured the correlation estimate between peaks per SCZ-specific CRDs) and used Mann-Whitney U test for comparing identified mean correlation estimates between SCZ cases and controls.

Enrichment of peaks within SCZ -specific CRDs for fetal- vs adult-identified chromatin peaks

To assess whether peaks within SCZ-specific CRDs are enriched for chromatin peaks detected in fetal vs adult DLPFC samples, we considered H3K27ac peaks captured within the PsychENCODE Human brain development resource(16). In PsychENCODE data, a peak was determined as fetal or adult if it was present in at least two of the three brain samples in one group of DLPFC samples and in none of the DLPFC samples of the other group. This resulted in 13,341 fetal-identified and 29,047 adult-identified peaks. As the PsychENCODE peaks were mapped to human genome assemble GRCh/hg38, we used UCSC Genome Browser liftOver tool(17) to convert the peak coordinates to the hg19 assembly. Next, we considered 40,819 peaks identified in the current study in SCZ cases only and determined peaks to be SCZ-specific if these belonged to a CRD that was composed of peaks not regrouping into any CRD in controls (6,650 peaks) and classified the rest to be shared (34,169 peaks). Then, we determined whether the SCZ-specific and shared peaks overlapped with PsychENCODE fetal or adult peaks and carried out enrichment analysis using Fisher's exact test (**Supplementary Tabel 3**). Sharing between peaks were determined by considering 50% reciprocal overlap between a CRD peak and a PsychENCODE peak using BEDTools v2.28.0(13).

CRD and gene association

Briefly, we considered normalized CRD activity quantifications (final step in CRD activity quantification section) and corrected and normalized gene expression quantifications and used QTLtools(6) cis permutation pass to identify CRDs associated with a gene in a +/-1 Mb window from a gene's transcription start site. We performed these analyses to i) identify genes associated with SCZ-specific CRDs, and ii) capture comparable associations for SCZ cases and controls using the same CRD annotations, i.e., CRDs identified in the combined set. The first approach was performed in SCZ cases only (n=59) using CRD activity quantifications retrieved based on the CRDs identified in SCZ cases (10,938 CRDs; **Supplementary Table 2, Supplementary Table 5)**. The second approach was done for SCZ cases (n=59), for controls (n=105) and across samples (n=164; disease status (SCZ/CTL) considered as a covariate) using CRD activity quantifications retrieved based on the CRDs identified in the combined set (11,374 CRDs; **Supplementary Table 1, Supplementary Table 9**).

Specifically, to capture technical and biological variability in gene expression data, we residualized for ancestry, using 3 genotype PCs, and for the number of optimal RNA-seq PCs that allowed to discover the maximum number of eQTLs. This was done similarly as for the ChIP-seq data by doing association testing at variable number of PCs. Gene expression quantifications were corrected for 3 genotype PCs and 10 RNA-seq PCs in SCZ cases, 3 genotype PCs and 30 RNA-seq PCs in controls, and 3 genotype PCs and 40 RNA-seq PCs in the combined set (**Supplementary Fig. 6d-f**). The resulting matrices were rank-normal transformed. Next, we enumerated all CRDs within +/-1 Mb of gene's transcription start site, tested their activity for association with gene expression and stored the best hit together with the nominal p-value. We adjusted the nominal p-value for the number of CRDs being tested in cis using permutation and corrected for the number of genes being tested using the R/*qvalue* package(15). We determined gene-CRD associations at FDR 5% as significant.

<u>Differential CRD activity and differential gene expression analysis</u>

Both differential CRD activity and differential gene expression analyses were carried out using DESeq2(18). Significant associations were determined at FDR 5% (**Supplementary Table 6, Supplementary Table 7**). For differential CRD activity analysis, we used unnormalized ChiP-seq read counts obtained with HOMER (annotatePeaks.pl with options -noann -nogene -size given -raw)(9) and summed these up per CRD using the ChiP-seq peak correlation map identified in the combined set (11,374 CRDs). For differential gene expression analysis, we used RNA-seq read counts. Unnormalized and raw sequencing counts is the requisite for using the DESeq2 pipeline for the applied statistical model to hold(18) as only count values allow correct assessment of measurement precision. Next, medians of ratios normalization was applied, whereby counts were divided by sample-size specific factors determined by median ratio of gene/peak counts relative to geometric per gene/peak. The median of ratios method assumes that not all genes/peaks are differentially expressed/active. Therefore, the normalization factors should account for sequencing depth and RNA/DNA composition of the sample (large outlier genes/peaks will not represent the median ratio values). This method is robust to imbalance in up- and down-regulation and large numbers of differentially expressed genes and active peaks.

To identify covariates for correction, we carried out association testing i) between all available biological and technical covariates and diagnosis status (Mann-Whitney U test), and ii) between all available biological and technical covariates and individual ChIP-seq peak activity and gene expression quantifications (linear regression) and calculated $\pi 1$ estimate(15) to identify the proportion of true associations. The technical and biological covariates we considered for differential gene expression analyses were as follows: age at death, genotype PC1, genotype PC2, genotype PC3, sex, brain weight, pH, post-mortem interval, total RNA yield, A260/A280 ratio, 15bp repeat in sequencing data, date of sequencing, library batch, empirical insert size, GC content in sequencing data, transcript integrity number, RNA integrity number. The technical and biological covariates we considered for differential CRD activity analysis were as follows: age at death, genotype PC1, genotype PC2, genotype PC3, sex, library batch, sequencing batch, flow cell, 15bp repeat in sequencing data, empirical insert size, GC content in sequencing data, sequencing lane. We identified the following covariates for differential gene expression analysis: sex, age at death, genotype PC1, genotype PC2, post-mortem interval, brain pH, brain weight, RNA integrity number, total RNA yield, A260/A280 ratio, GC content in sequencing data, transcript integrity number,

empirical insert size, 15bp repeat in sequencing data, date of sequencing. We identified the following covariates for differential CRD activity analysis: sex, age at death, genotype PC1, library batch, GC content in sequencing data, empirical insert size, 15bp repeat in sequencing data.

To identify whether differentially expressed genes (DEGs) and differentially active CRDs (DACs) overlap, we considered two different approaches: i) based on genomic position, ii) based on CRD-gene associations. In the former approach we asked whether the TSS of DEG lies within DAC more often than expected by chance. In the latter approach we first correlated gene expression and CRD activity using linear regression and then asked whether DEGs and DACs show higher correlation compared to non-DEGs and non-DACs.

R/*clusterProfiler* package(19) was used for gene set enrichment analysis. We considered genes that were either i) significantly differentially expressed (regardless of direction of effect), ii) significantly down-regulated or iii) significantly up-regulated. Significant associations were determined at FDR 5% (**Supplementary Table 8**).

Enrichment analysis of CRD peaks for SCZ GWAS variants

To estimate the enrichment of SCZ GWAS variants in differentially active CRDs and in SCZ-specific CRDs, two complementary approaches were used: MAGMA v1.10(20) and partitioned LD score regression v1.0.1(21). The former approach implemented as a linear regression model on peak-level data, accounting for LD between peaks, was used to test i) whether ChIP-seq peaks within SCZ-specific CRDs were more enriched for SCZ GWAS variants compared to ChIP-seq peaks within CRDs that were shared with controls, ii) whether ChIP-seq peaks within differentially active CRDs (DACs) showed greater association compared to non-DAC peaks, and iii) whether ChIP-seq peaks within significantly up-regulated DACs showed greater enrichment compared to those within significantly down-regulated DACs. Partitioned LD score regression allows to estimate whether SNV heritability for phenotype of interest is enriched for pre-defined genomic features against the baseline model and accounting for genomic LD structure. This approach was used to compute SCZ heritability in enrichment for peaks within DACs, for peaks either in up-regulated or down-regulated DACs, and for peaks within SCZ-specific and within shared CRDs. For both analyses summary statistics from SCZ GWAS wave3(22) (excluding the major histocompatibility complex region and default parameters were used. All considered sets of peaks covered more than 0.05% of the human genome.

Association of aCRD-QTLs and eQTLs with the other molecular phenotype

We tested QTL effects for association with the other molecular phenotype (i.e., gene expression with aCRD-QTLs and CRD activity with eQTLs) via CRD-gene nominal associations using CRD identified in the combined set. Specifically, for each gene we identified in cis window all associated CRDs at nominal pass, and for each CRD determined in cis window all associated genes at nominal pass. Using these intermediate associations, we could look whether e.g., an eQTL also affects the CRD that the targeted eGene is associated with, and vice versa, whether the aCRD-QTL affects the gene that the impacted aCRD is associated with. Proportion of sharing was estimated using $\pi1$ estimate(15).

Colocalization with SCZ GWAS variants

Briefly, we assessed the likelihood of a shared functional effect between independent SCZ risk variants from four GWAS studies(22-25) and SCZ-identified QTLs (857 aCRD-QTLs and 987 eQTLs) using regulatory trait concordance (RTC). This algorithm assesses the likelihood of a shared functional effect between a GWAS variant and a QTL variant by quantifying the change in the statistical significance of the QTL after correcting the QTL phenotype (gene expression or CRD activity) for the genetic effect of the GWAS variant and comparing its correction impact to that of all other SNPs in the interval(26, 27). We applied a cut-off of RTC $\geq$0.9 for determining a shared functional effect. The output files indicate the union results across four GWAS studies. When accounting for LD, we see 16 independent shared effects between GWAS and eQTL variants and 11 independent shared effects between GWAS and aCRD-QTL variants (**Supplementary Table 13**).

CRD-gene pair quantification

To quantify the 1,197 gene-CRD pairs we identified as significant at FDR 5% across SCZ cases and controls (n=164), we used PCA-based dimensionality reduction. For each gene-CRD pair, we aggregated gene expression with CRD activity and used the coordinates on PC1 as new pseudo-phenotypes. For identifying genetic variants that affect both the CRD activity and the gene expression (per gene-CRD pair), we used the new derived pseudo-phenotypes and carried out an eCRD-QTL (genetic variant that affects both the CRD and gene) discovery analysis in cis across all samples (n=164) using permutation.

Causal inference for determining causal relationships for eQTLCRD-CRD-gene triplets

We applied a Bayesian Network approach to infer the most likely causal relationship for eCRDQTL-CRD-gene triplets common to SCZ cases and controls (1,134 triplets) and conducted the analyses separately in SCZ cases (n=59) and in controls (n=105). This approach allowed to estimate the most likely network from which the observed data originates. The starting point is always the genetic variant as this does not change (genome is fixed). We explored three distinct models (topologies): i) causal model in which the genetic variant affects first the CRD and then the gene, ii) reactive model in which the genetic variant affects the gene and then the CRD, iii) independent model in which the genetic variant affects the gene and the CRD independently (**Supplementary Fig. 22**). For each triplet we built a 59 x 3 and 105 x 3 data matrix for SCZ cases and controls, respectively, that contained normalized quantifications, and calculated the likelihood of three possible Bayesian Network topologies using R/*bnlearn* package(28). We converted the likelihoods to posterior probabilities, assuming a uniform prior probability for three possible models.

Bootstrapping

To estimate the accuracy for the Bayesian Network results and provide confidence for retrieved probabilities, we used bootstrapping. We carried out 100 bootstrapping runs for each tested triplet separately for SCZ cases (n=59) and controls (n=105) using sampling with replacement. For accuracy estimation, we calculated how many times the most probable model across bootstrapping runs for each triplet was the same as in the original Bayesian Network results. We filtered out all triplets that fell below a confidence threshold of 55%: this corresponds to the lower quartile value in SCZ cases (**Supplementary Fig. 24ab**).

Gene set enrichment for model-change associated triplets

R/*clusterProfiler* package(19) was used for gene set enrichment analysis for genes that belonged to triplets showing directional change from eCRD-QTL onto gene expression/CRD activity between SCZ cases and controls. We considered two scenarios: i) causal model in controls, but reactive/independent in SCZ cases (n=130); ii) reactive/independent in controls, but causal in SCZ cases (n=98).

REFERENCES

1. Lipska BK, Deep-Soboslay A, Weickert CS, Hyde TM, Martin CE, Herman MM, et al. Critical factors in gene expression in postmortem human brain: Focus on studies in schizophrenia. *Biol Psychiatry*. 2006; **60**: 650-8.
2. Chang CC, Chow CC, Tellier LC, Vattikuti S, Purcell SM, Lee JJ. Second-generation PLINK: rising to the challenge of larger and richer datasets. *Gigascience*. 2015; **4**: 7.
3. Taliun D, Harris DN, Kessler MD, Carlson J, Szpiech ZA, Torres R, et al. Sequencing of 53,831 diverse genomes from the NHLBI TOPMed Program. *Nature*. 2021; **590**: 290-9.
4. Altshuler DM, Durbin RM, Abecasis GR, Bentley DR, Chakravarti A, Clark AG, et al. A global reference for human genetic variation. *Nature*. 2015; **526**: 68-74.
5. Dobin A, Davis CA, Schlesinger F, Drenkow J, Zaleski C, Jha S, et al. STAR: ultrafast universal RNA-seq aligner. *Bioinformatics*. 2013; **29**: 15-21.
6. Delaneau O, Ongen H, Brown AA, Fort A, Panousis NI, Dermitzakis ET. A complete tool set for molecular QTL discovery and analysis. *Nat Commun*. 2017; **8**: 15452.
7. Li H. Aligning sequence reads, clone sequences and assembly contigs with BWA-MEM. Preprint at arXiv:13033997v2. 2013.
8. Kharchenko PV, Tolstorukov MY, Park PJ. Design and analysis of ChIP-seq experiments for DNA-binding proteins. *Nat Biotechnol*. 2008; **26**: 1351-9.

9. Heinz S, Benner C, Spann N, Bertolino E, Lin YC, Laslo P, et al. Simple combinations of lineage-determining transcription factors prime cis-regulatory elements required for macrophage and B cell identities. *Mol Cell*. 2010; **38**: 576-89.
10. Fort A, Panousis NI, Garieri M, Antonarakis SE, Lappalainen T, Dermitzakis ET, et al. MBV: a method to solve sample mislabeling and detect technical bias in large combined genotype and sequencing assay datasets. *Bioinformatics*. 2017; **33**: 1895-7.
11. Delaneau O, Zazhytska M, Borel C, Giannuzzi G, Rey G, Howald C, et al. Chromatin three-dimensional interactions mediate genetic effects on gene expression. *Science*. 2019; **364**: eaat8266.
12. Wang DF, Liu S, Warrell J, Won H, Shi X, Navarro FCP, et al. Comprehensive functional genomic resource and integrative model for the human brain. *Science*. 2018; **362**.
13. Quinlan AR, Hall IM. BEDTools: a flexible suite of utilities for comparing genomic features. *Bioinformatics*. 2010; **26**: 841-2.
14. Girdhar K, Hoffman GE, Bendl J, Rahman S, Dong P, Liao W, et al. Chromatin domain alterations linked to 3D genome organization in a large cohort of schizophrenia and bipolar disorder brains. *Nat Neurosci*. 2022; **25**: 474-83.
15. Storey JD, Tibshirani R. Statistical significance for genomewide studies. *Proc Natl Acad Sci U S A*. 2003; **100**: 9440-5.
16. Li M, Santpere G, Imamura Kawasawa Y, Evgrafov OV, Gulden FO, Pochareddy S, et al. Integrative functional genomic analysis of human brain development and neuropsychiatric risks. *Science*. 2018; **362**.
17. Hinrichs AS, Karolchik D, Baertsch R, Barber GP, Bejerano G, Clawson H, et al. The UCSC Genome Browser Database: update 2006. *Nucleic Acids Res*. 2006; **34**: D590-8.
18. Love MI, Huber W, Anders S. Moderated estimation of fold change and dispersion for RNA-seq data with DESeq2. *Genome Biol*. 2014; **15**: 550.
19. Yu G, Wang LG, Han Y, He QY. clusterProfiler: an R package for comparing biological themes among gene clusters. *OMICS*. 2012; **16**: 284-7.
20. de Leeuw CA, Mooij JM, Heskes T, Posthuma D. MAGMA: generalized gene-set analysis of GWAS data. *PLoS Comput Biol*. 2015; **11**: e1004219.
21. Finucane HK, Bulik-Sullivan B, Gusev A, Trynka G, Reshef Y, Loh PR, et al. Partitioning heritability by functional annotation using genome-wide association summary statistics. *Nature Genet*. 2015; **47** :1228-35.
22. Trubetskoy V, Pardinas AF, Qi T, Panagiotaropoulou G, Awasthi S, Bigdeli TB, et al. Mapping genomic loci implicates genes and synaptic biology in schizophrenia. *Nature*. 2022; **604**: 502-8.
23. Schizophrenia Working Group of the Psychiatric Genomics Consortium. Biological insights from 108 schizophrenia-associated genetic loci. *Nature*. 2014; **511**: 421-7.
24. Pardinas AF, Holmans P, Pocklington AJ, Escott-Price V, Ripke S, Carrera N, et al. Common schizophrenia alleles are enriched in mutation-intolerant genes and in regions under strong background selection. *Nat Genet*. 2018; **50**: 381-9.
25. Bigdeli TB, Genovese G, Georgakopoulos P, Meyers JL, Peterson RE, Iyegbe CO, et al. Contributions of common genetic variants to risk of schizophrenia among individuals of African and Latino ancestry. *Mol Psychiatry*. 2020; **25**: 2455-67.
26. Ongen H, Brown AA, Delaneau O, Panousis NI, Nica AC, Consortium GT, et al. Estimating the causal tissues for complex traits and diseases. *Nat Genet*. 2017; **49**: 1676-83.
27. Nica AC, Montgomery SB, Dimas AS, Stranger BE, Beazley C, Barroso I, et al. Candidate Causal Regulatory Effects by Integration of Expression QTLs with Complex Trait Genetic Associations. *Plos Genet*. 2010; **6**.
28. Scutari M. Learning Bayesian Networks with the bnlearn R Package. *J Stat Softw*. 2010; **35** :1-22.

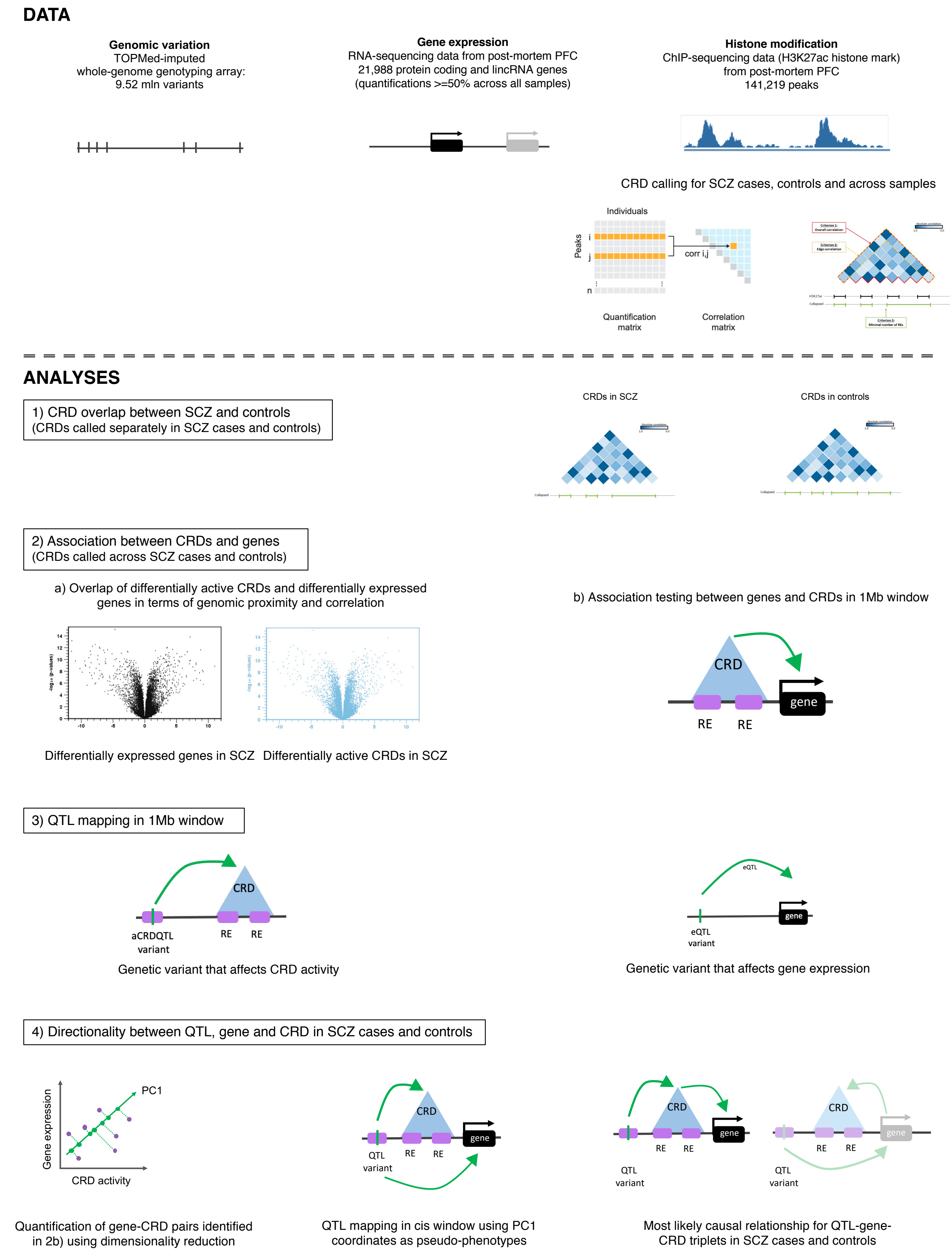

Supplementary Fig. 1. Schematic overview of the data used, and analyses conducted.

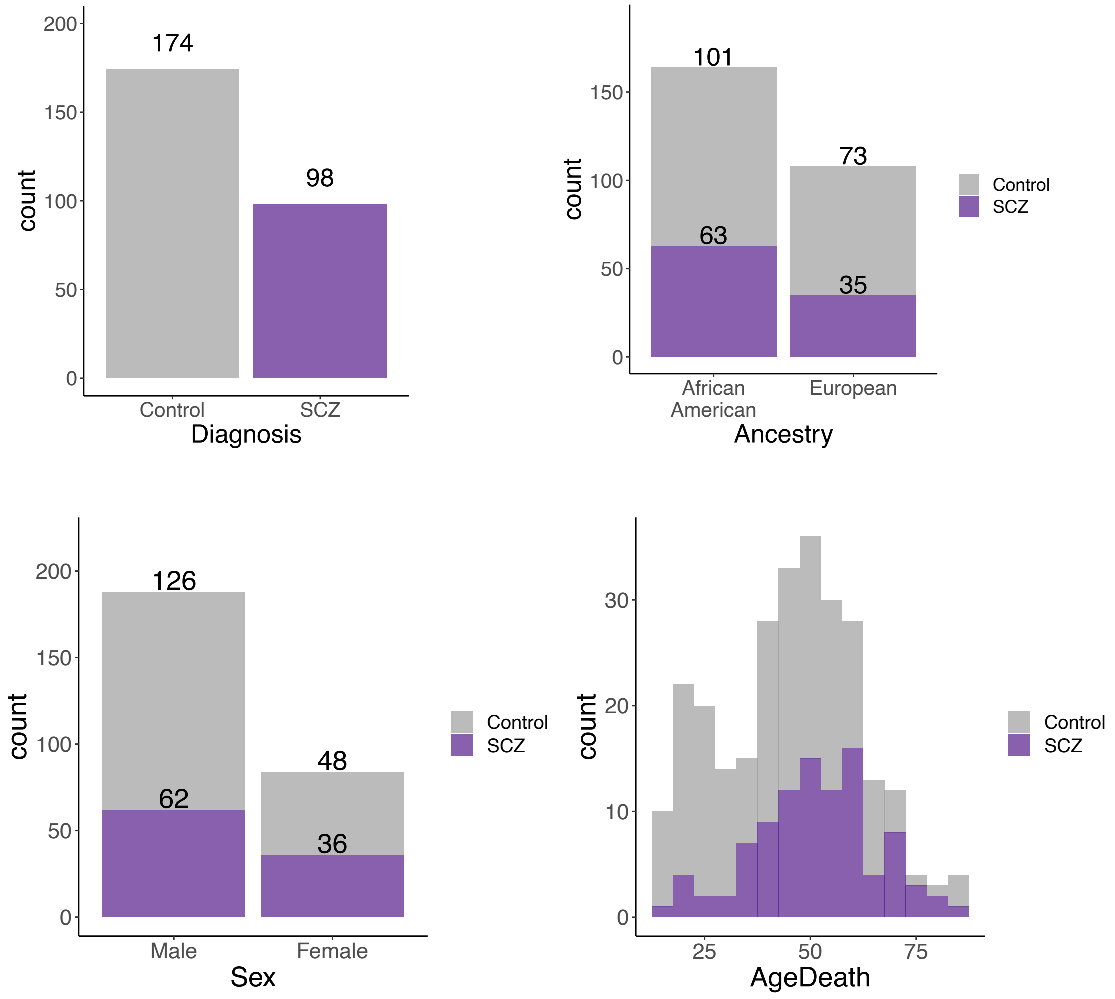

Supplementary Fig. 2. Overview of the phenotypic characteristics of the HBCC cohort. Mean age 51 years (sd = 14.4) for schizophrenia (SCZ) cases and 42.2 years (sd = 16.4) for controls.

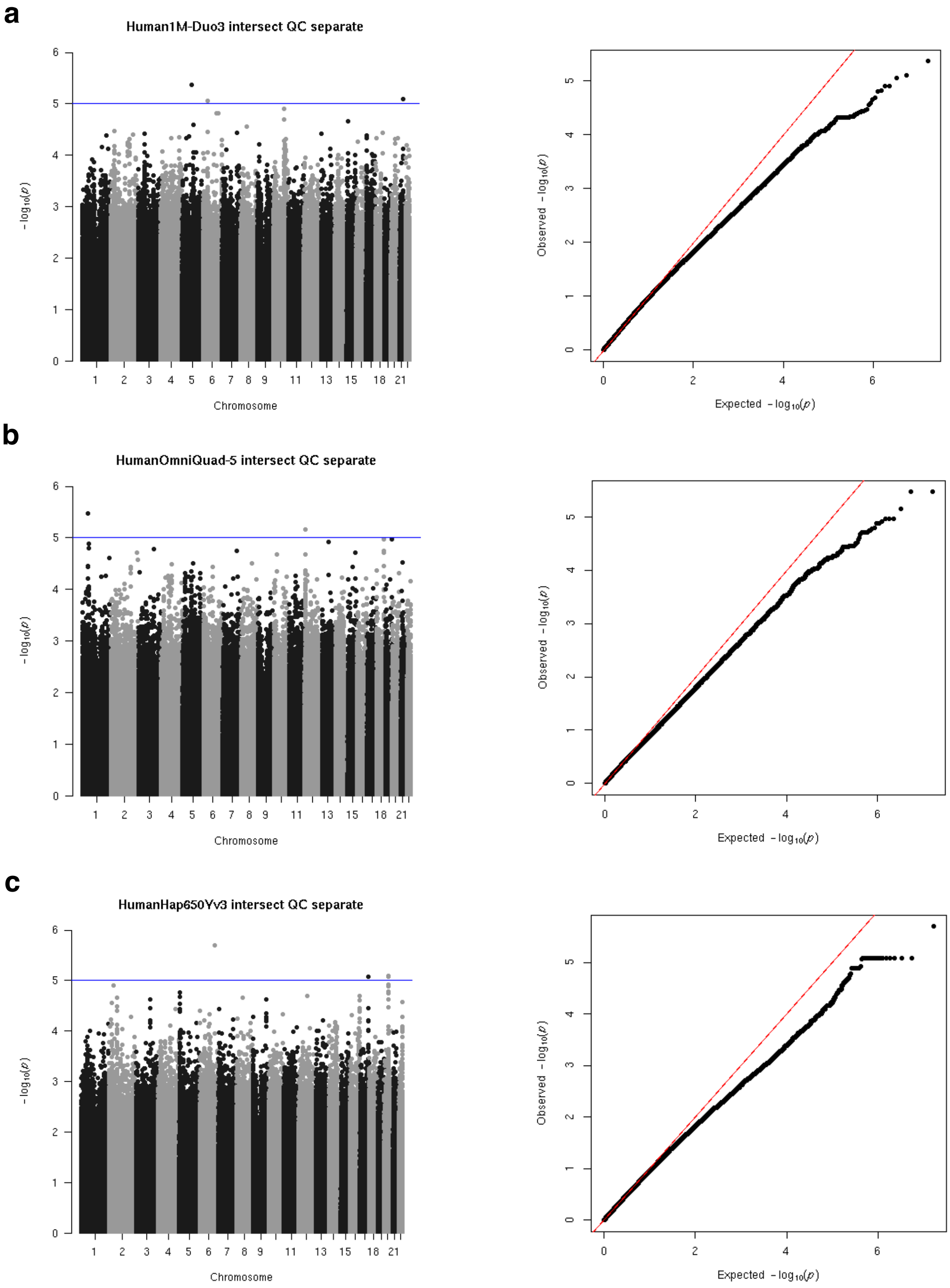

Supplementary Fig. 3. Association testing between imputed SNVs and genotyping array. For each genotyping array, an association test was carried out using logistic regression for array of interest (either (a) Illumina Human1M-Duov3, (b) Illumina HumanOmni5M-Quad or (c) Illumina HumanHap650Y) vs other two arrays. Manhattan and QQ-plots depict the distribution of imputed SNVs per chromosome as a function of -log10 p-values and expected vs observed p-values, respectively. Post-imputation quality control (MAF >0.05 and HWE >1e-6) was applied by ancestry and the union SNV content across ancestry sets was considered for this and all downstream analyses. No bias towards any genotyping arrays was detected.

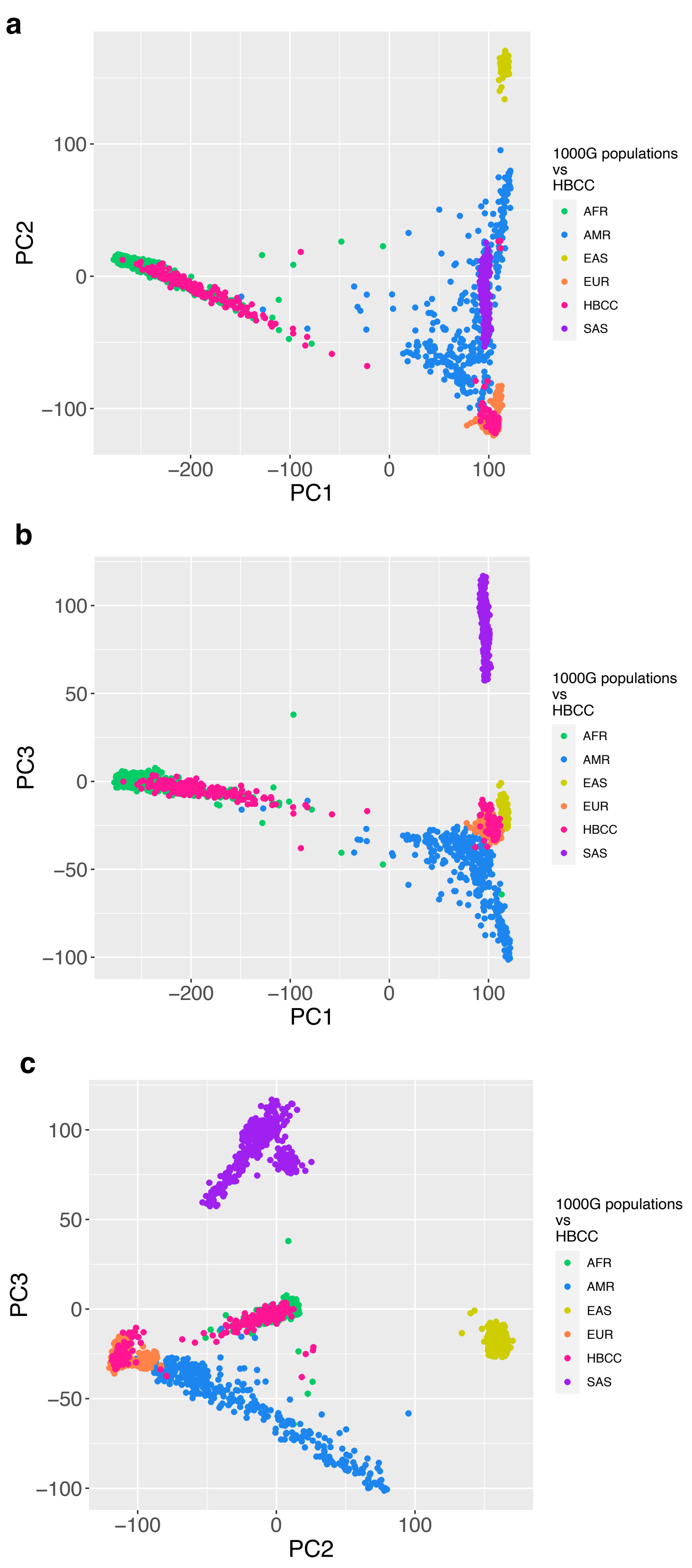

Supplementary Fig. 4. Principal component (PC) analysis of genotype data. Coordinates of (a) PC1 vs PC2, (b) PC1 vs PC3 and (c) PC2 vs PC3 in reference to the 1000 Genome Project samples. HBCC cohort samples are coloured in pink and cluster at PC coordinates represented by the African and European 1000 Genome Project super populations.

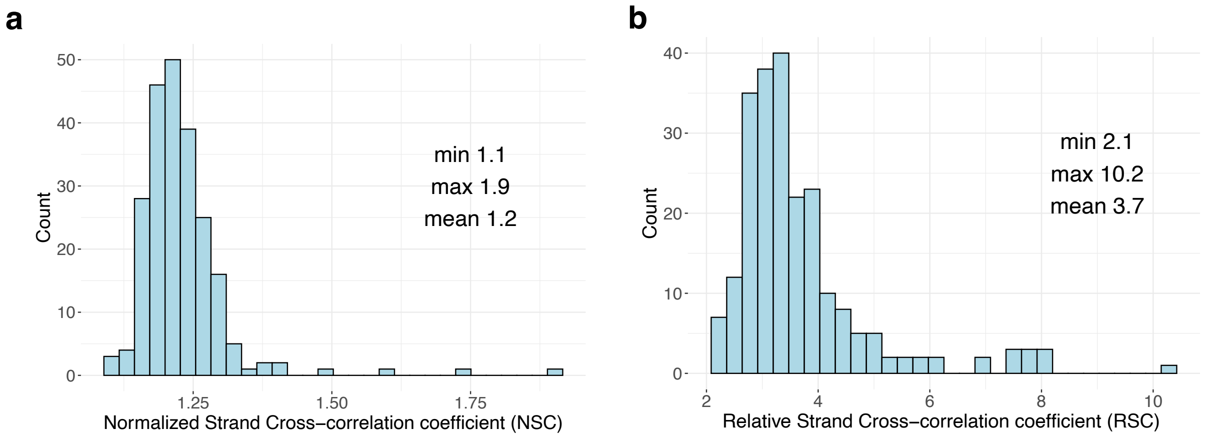

Supplementary Fig. 5. PhantomPeakQualTools quality metrics. (a) Normalized Strand Cross-Correlation coefficient (NSC) distribution and (b) Relative Strand Cross-correlation coefficient (RSC) distribution for H3K27ac peaks in HBCC samples. NSC <1.05 and RSC <0.8 indicate low signal to noise.

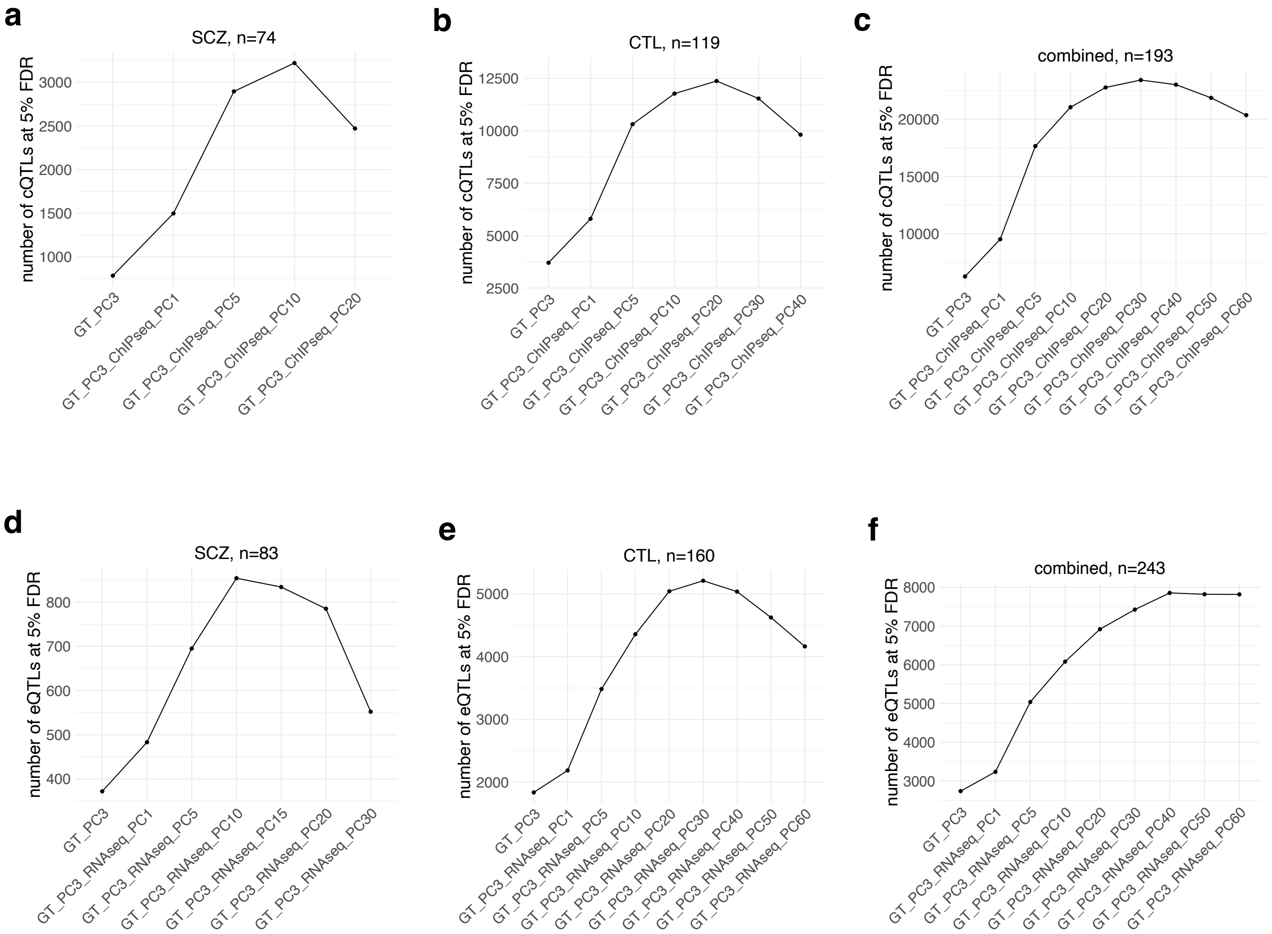

Supplementary Fig. 6. Optimization of QTL discovery. Number of chromatin-QTLs (cQTLs) and expression-QTLs (eQTLs) discovered (a,d) in SCZ cases, (b,e) in controls (CTL) and (c,f) in the combined set as a function of the number of genotype (GT) principal components (PCs) and ChIP-seq and RNA-seq PCs used to residualize ChIP-seq peak quantification and gene expression quantification data, respectively. The PCs that allowed the discovery of the maximum number of QTLs were retrained for downstream analyses.

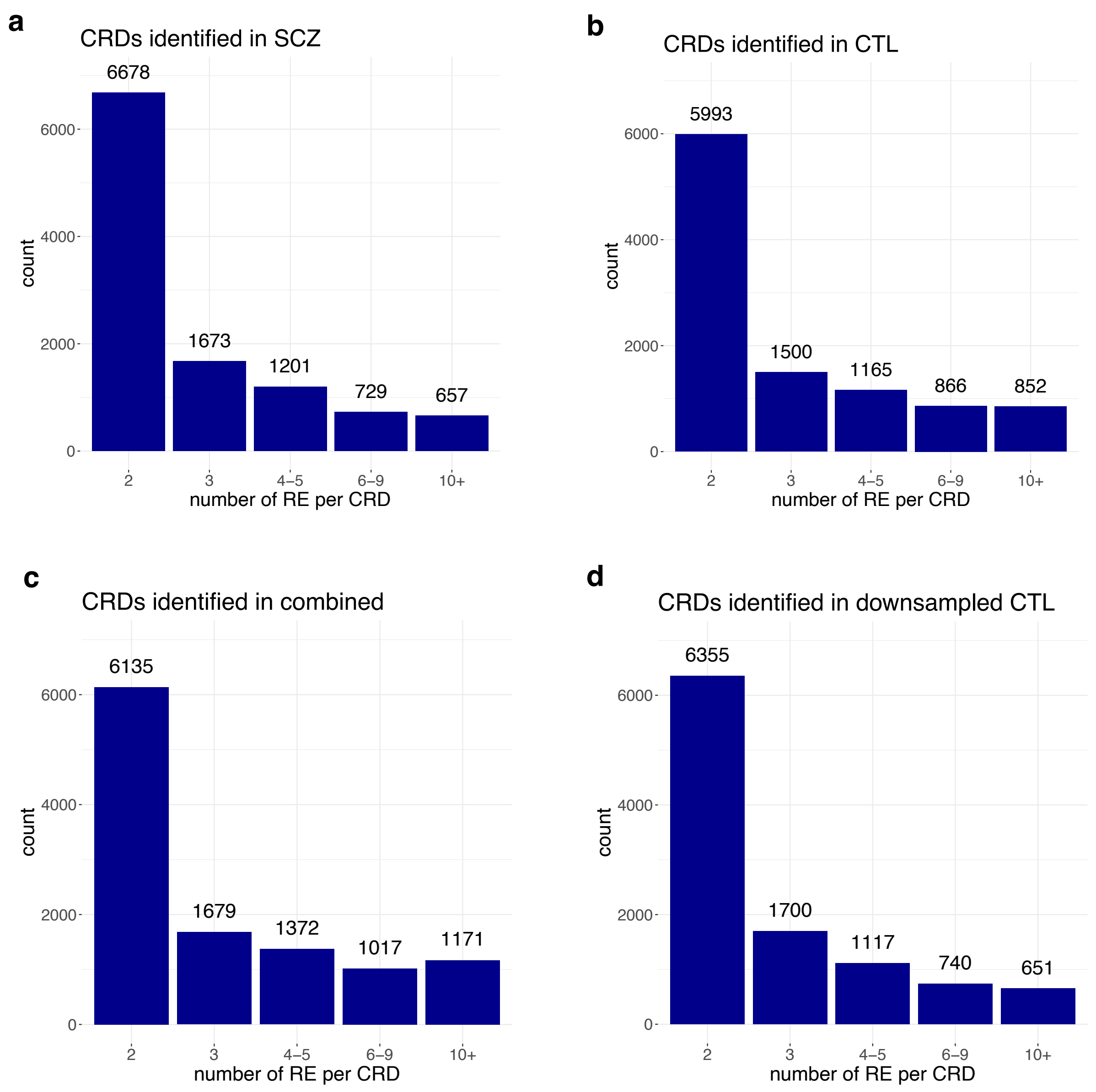

Supplementary Fig. 7. Regulatory element (RE) content of CRDs. Number of regulatory elements per CRD (a) in SCZ cases (n=74), (b) in controls (CTL, n=119), (c) in the combined set (n=193) and (d) in down-sampled controls (n=74). Mean number of REs per CRD was 3.7, 4.3, 4.7 in SCZ cases, in controls, in the combined set, respectively. Mean CRD length was 137,017 bp, 135,734 bp and 138,144 base pairs in SCZ cases, in controls and in the combined set, respectively.

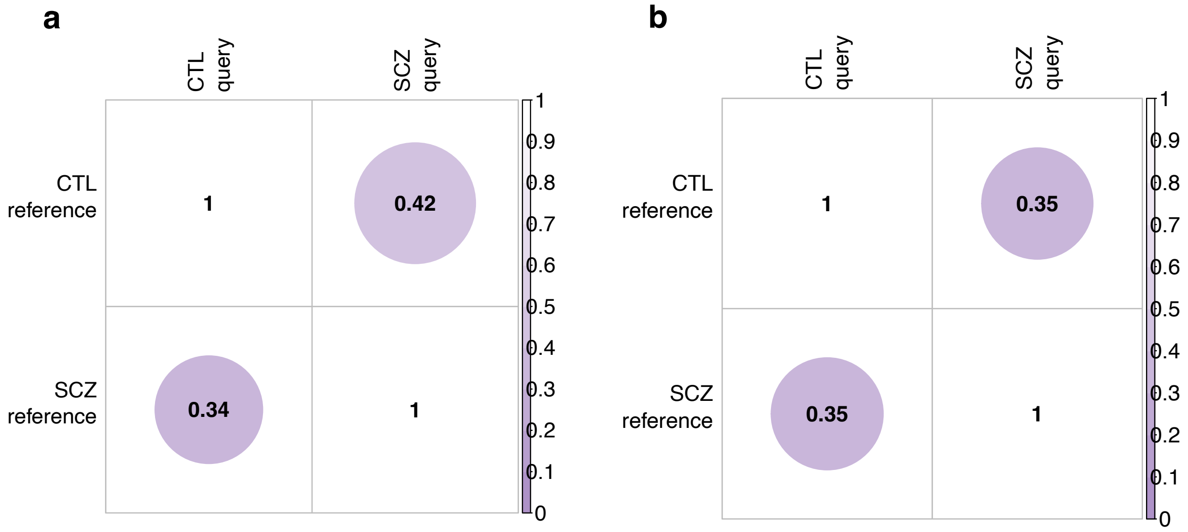

Supplementary Fig. 8. Fraction of CRD peak content sharing between SCZ cases and controls (CTL). A CRD was deemed shared between SCZ cases and controls in case ≥50% of ChIP-seq peaks of the reference CRD were present in a CRD from the query state. (a) Forty-two percent of CRDs detected in controls (n=119) were also detected in SCZ cases (n=74) and vice versa, 34% of CRDs detected in SCZ cases were also detected in controls. (b) CRD sharing between SCZ cases and down-sampled controls. Thirty-five percent of CRDs detected in down-sampled controls (n=74) were also detected in SCZ cases (n=74) and vice versa.

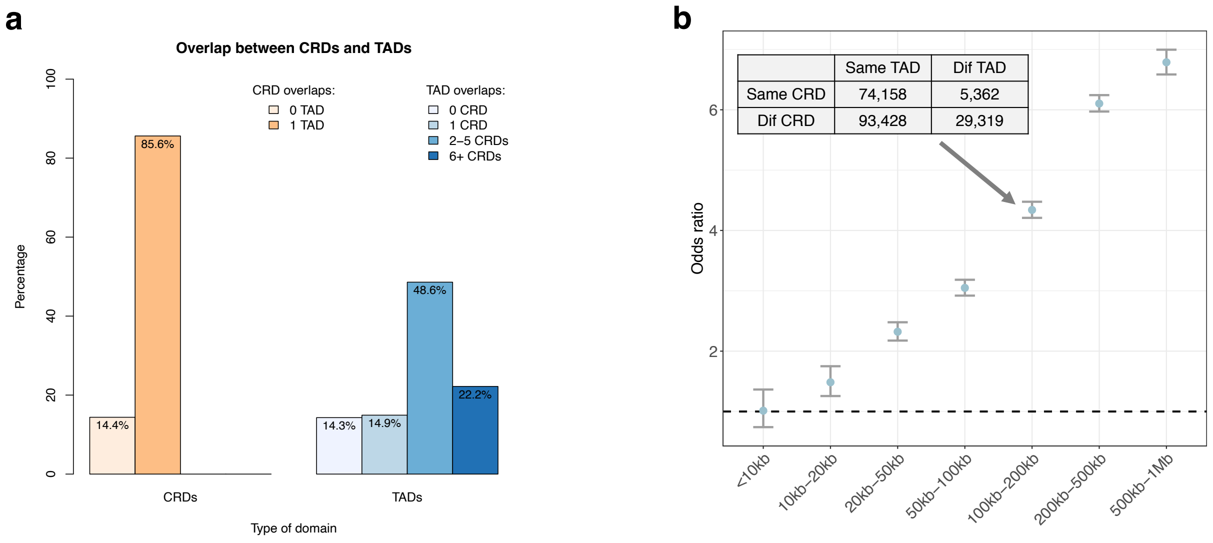

Supplementary Fig. 9. Overlap between CRDs and TADs. (a) Percentages of CRDs and TADs that overlap stratified by the number of overlapping features. (b) Odds ratios for pairs of peaks in the same CRD to also fall within the same TAD, measured in multiple distance bins. A contingency table example from which the odds ratios were computed is shown.

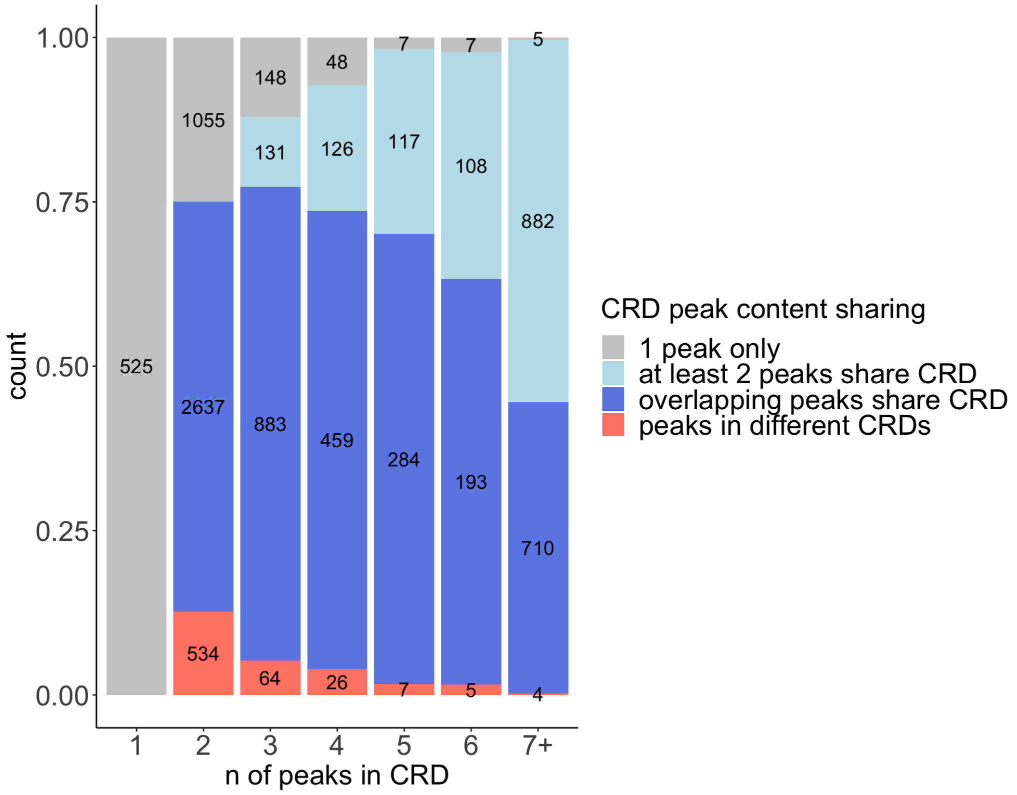

Supplementary Fig. 10. Overlap between CRD peaks with those detected in Girdhar et al.(14). The plot visualizes whether the overlapping peaks that form CRDs in both studies (43,501 peaks in 8,965 CRDs) cluster similarly into CRDs. The x-axis displays the number of peaks per CRD in our data and the colours indicate the proportion of CRD peak content sharing with Girdhar et al.: grey indicates that only one peak within a CRD in both datasets overlap, light blue indicates that at least two overlapping peaks cluster into the same CRD in Girdhar et al. data, dark blue denotes that all overlapping peaks per CRD in our data cluster into the same CRD in Girdhar et al. data, and red indicates that peaks cluster into different CRDs.

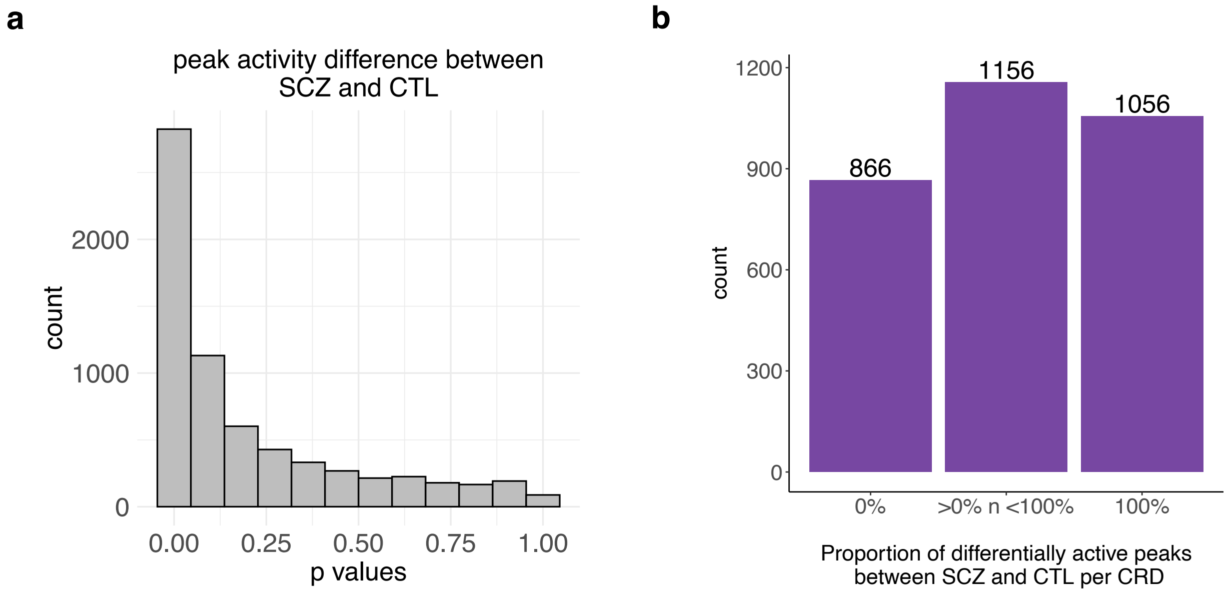

Supplementary Fig. 11. Differential peak activity between SCZ cases and controls (CTL). Only peaks clustering into SCZ-specific CRDs (i.e., CRDs composed of peaks not part of any CRD in controls) were considered. (a) P-value distribution of peaks differentially active between SCZ cases and controls. (b) Proportion of differentially active peaks (3,540 peaks) between SCZ cases and controls per SCZ-specific CRDs at FDR 5%. One-third of SCZ-specific CRDs (1,056 CRDs and 2,242 peaks) were forming due to all underlying peaks showing significantly different peak activity in SCZ cases compared to controls.

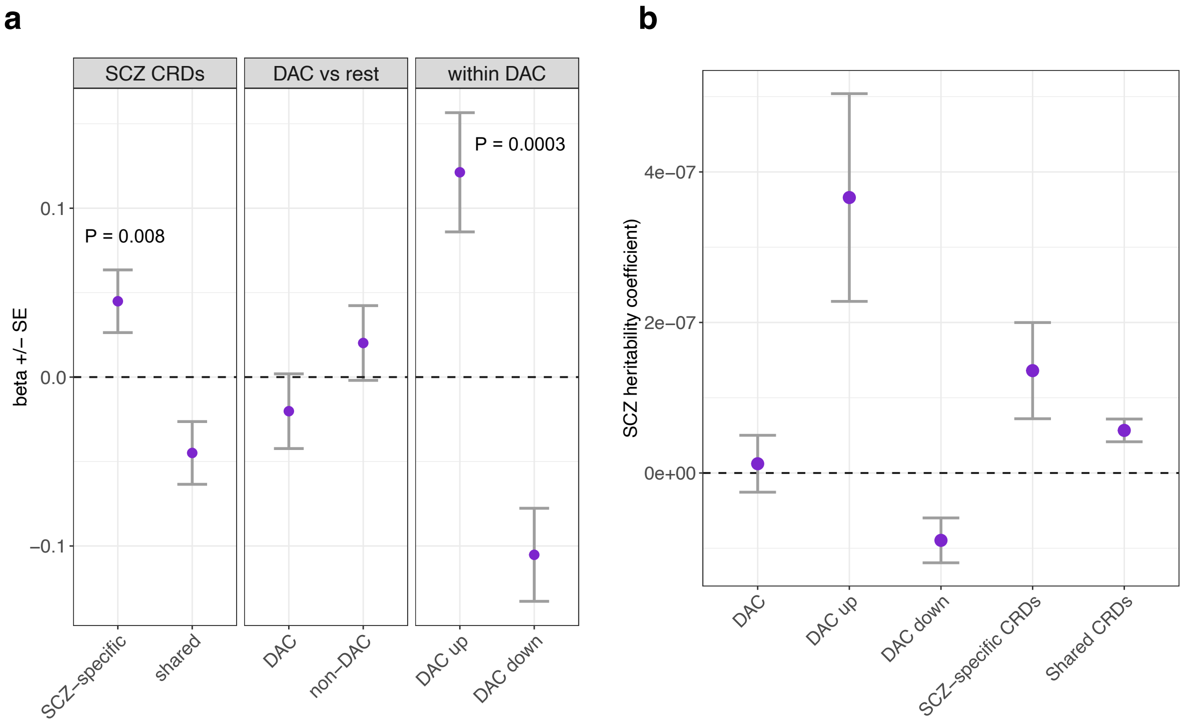

Supplementary Fig. 12. Enrichment of SCZ GWAS variants in SCZ-identified CRD peaks and differentially active CRDs peaks. (a) Coefficients of enrichment of SCZ GWAS variants using MAGMA for 1) peaks regrouping into CRDs only in SCZ cases compared to peaks in CRDs shared with controls, 2) for peaks regrouping into CRDs that were significantly differentially active compared to controls vs those that were not, 3) for peaks that clustered into CRDs that were significantly up-regulated in SCZ cases vs those that were significantly down-regulated. (b) SCZ heritability coefficients of SCZ GWAS variants detected using partitioned LD score regression for peaks within DACs, in up-regulated and down-regulated DACs, within SCZ-specific CRDs and within shared CRDs.

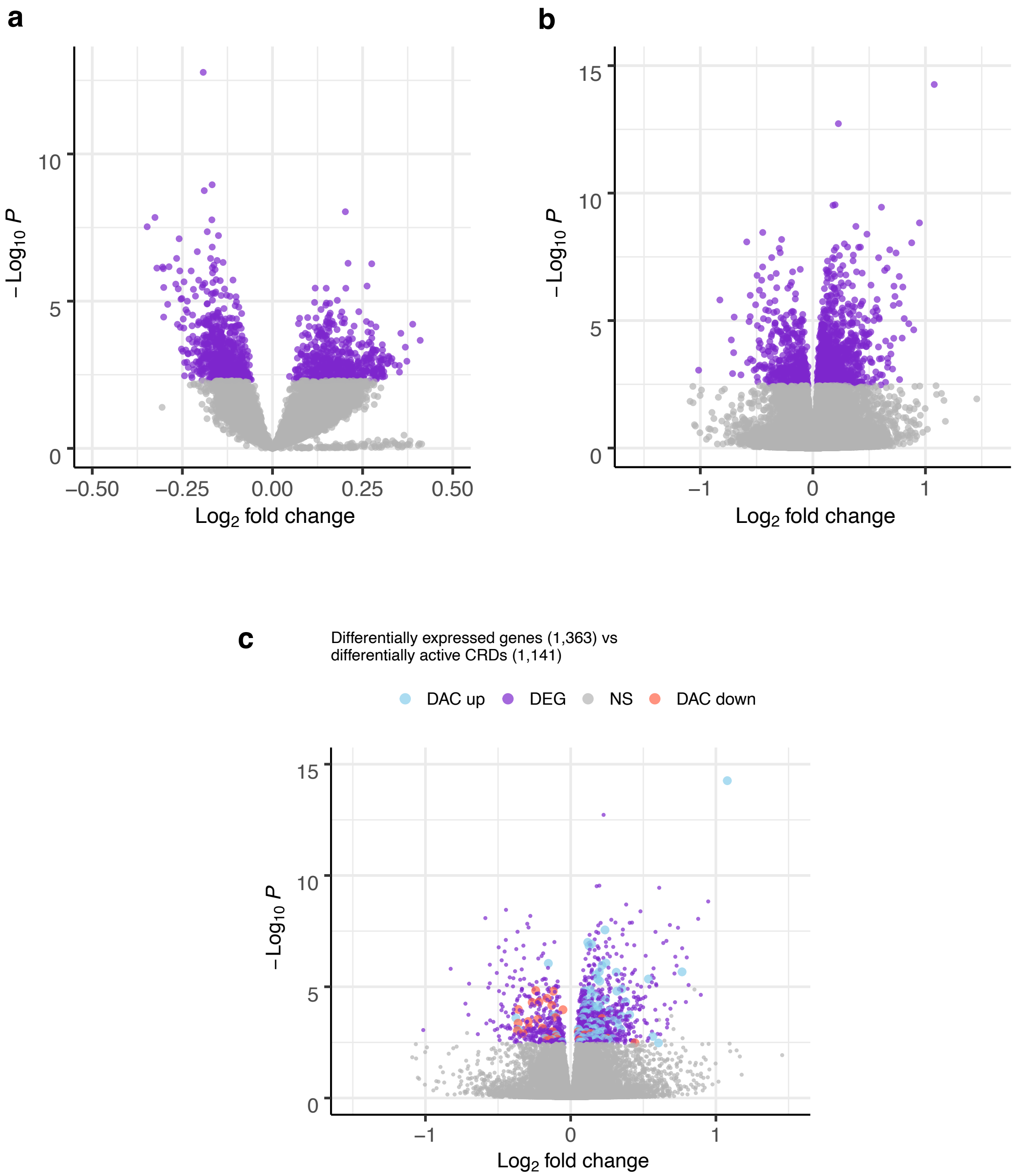

Supplementary Fig. 13. Volcano plots outlining (a) differential CRD activity and (b) differential gene expression between SCZ cases and controls. Differentially active CRDs genome-wide (n=1,141; 599 with lower activity and 542 with higher activity) and differentially expressed genes genome-wide (n=1,363; 937 up-regulated and 426 down-regulated), respectively, at FDR 5% are highlighted in purple. (c) TSSs of differentially expressed genes (DEG) were localized within differentially active CRDs (DACs) significantly more often than expected by chance (Fischer's exact test p = 8.72×10^{-6}, odds ratio 1.60); coloured dots denote DEGs genome-wide identified at FDR 5%: purple dots mark DEGs, blue dots denote DEGs with TSS within DAC with concordant effect direction, red dots mark DEGs with TSS within DAC with discordant effect direction.

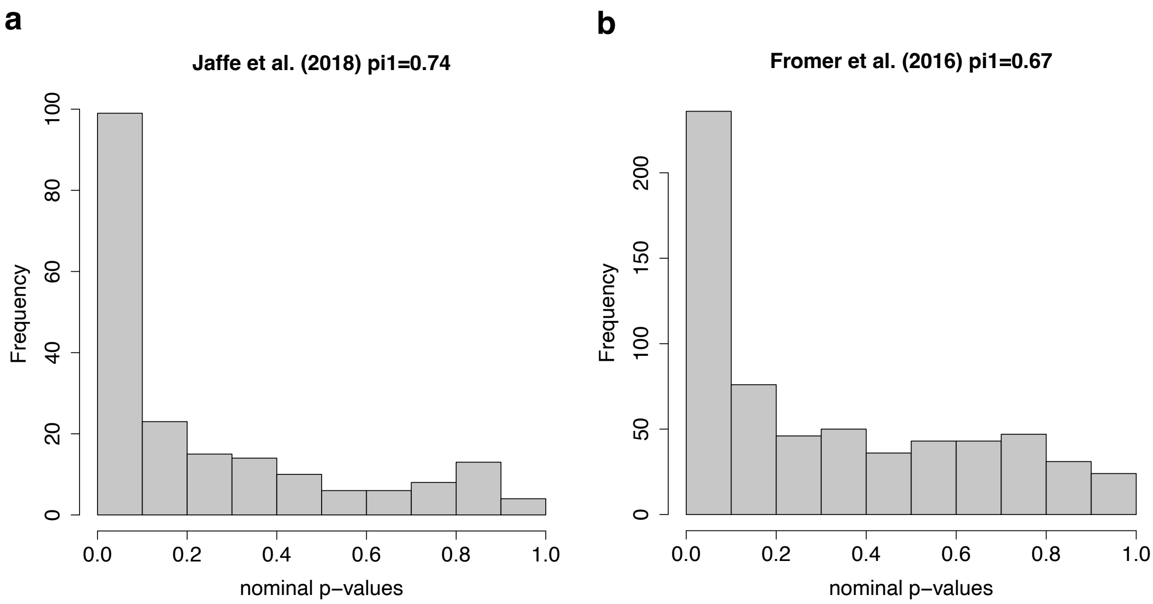

Supplementary Fig. 14. Replication of differential gene expression analysis results for SCZ in previously published findings. Differentially expressed genes identified at FDR 5% were in concordance with findings published by (a) Jaffe et al., 2018 and by (b) Fromer et al., 2016 based on π1 estimate.

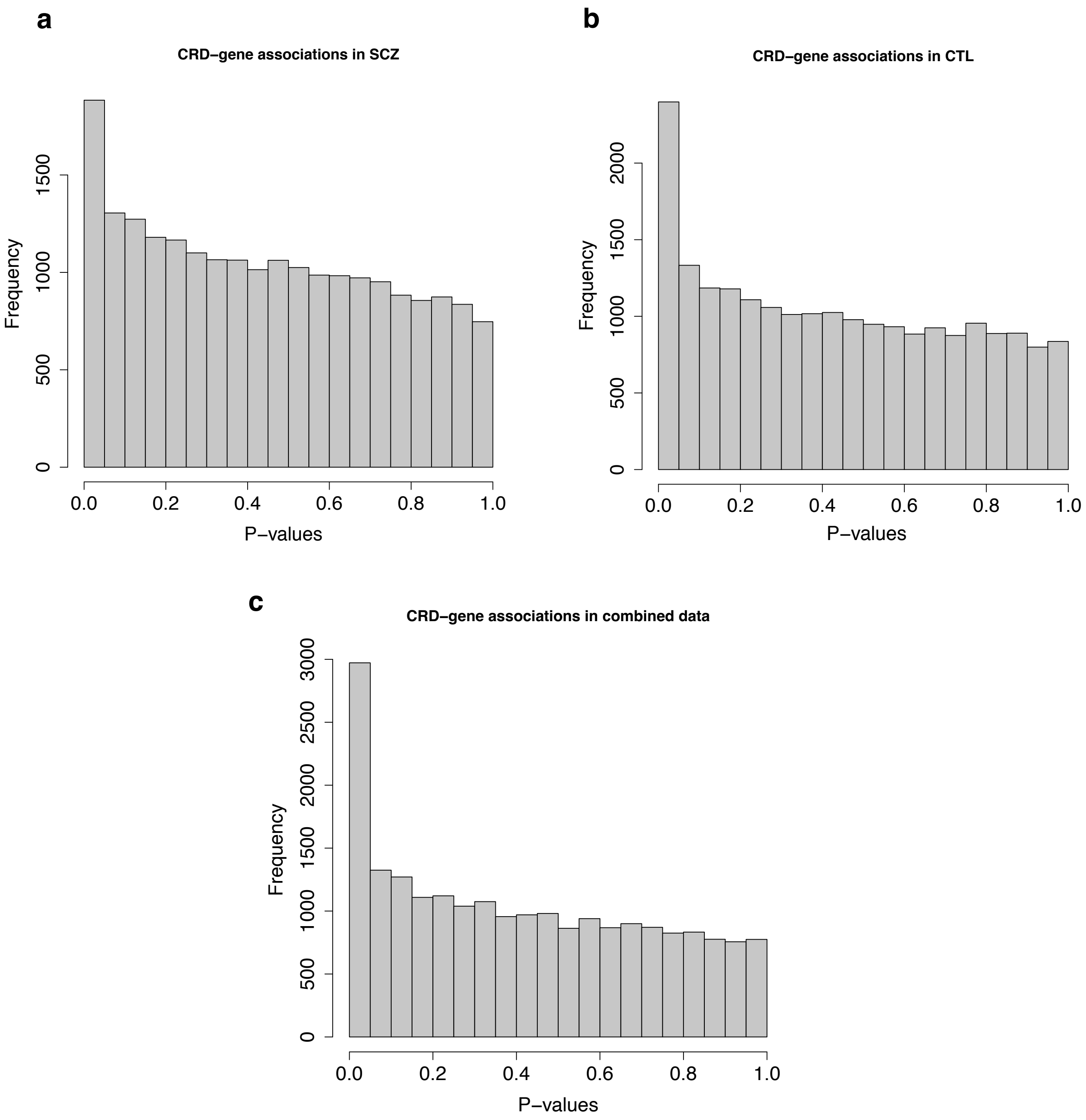

Supplementary Fig. 15. Gene-CRD associations. P-value distribution for gene-CRD associations (a) in SCZ cases (n=59), (b) in controls (n=105) and (c) in the combined set (n=164). At FDR 5%, 95, 634 and 1,197 CRD-gene associations were identified in SCZ cases, in controls and in the combined set, respectively.

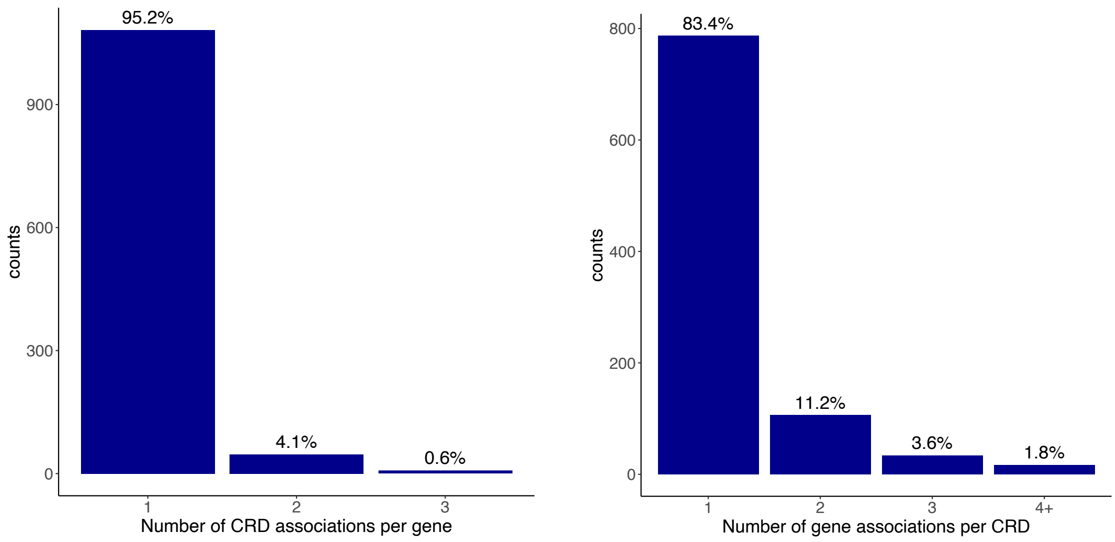

Supplementary Fig. 16. Gene-CRD associations. Number of genes and CRDs as a function of the number of CRDs and genes they were associated with, respectively, identified in the combined set (n=1,197 gene-CRD associations).

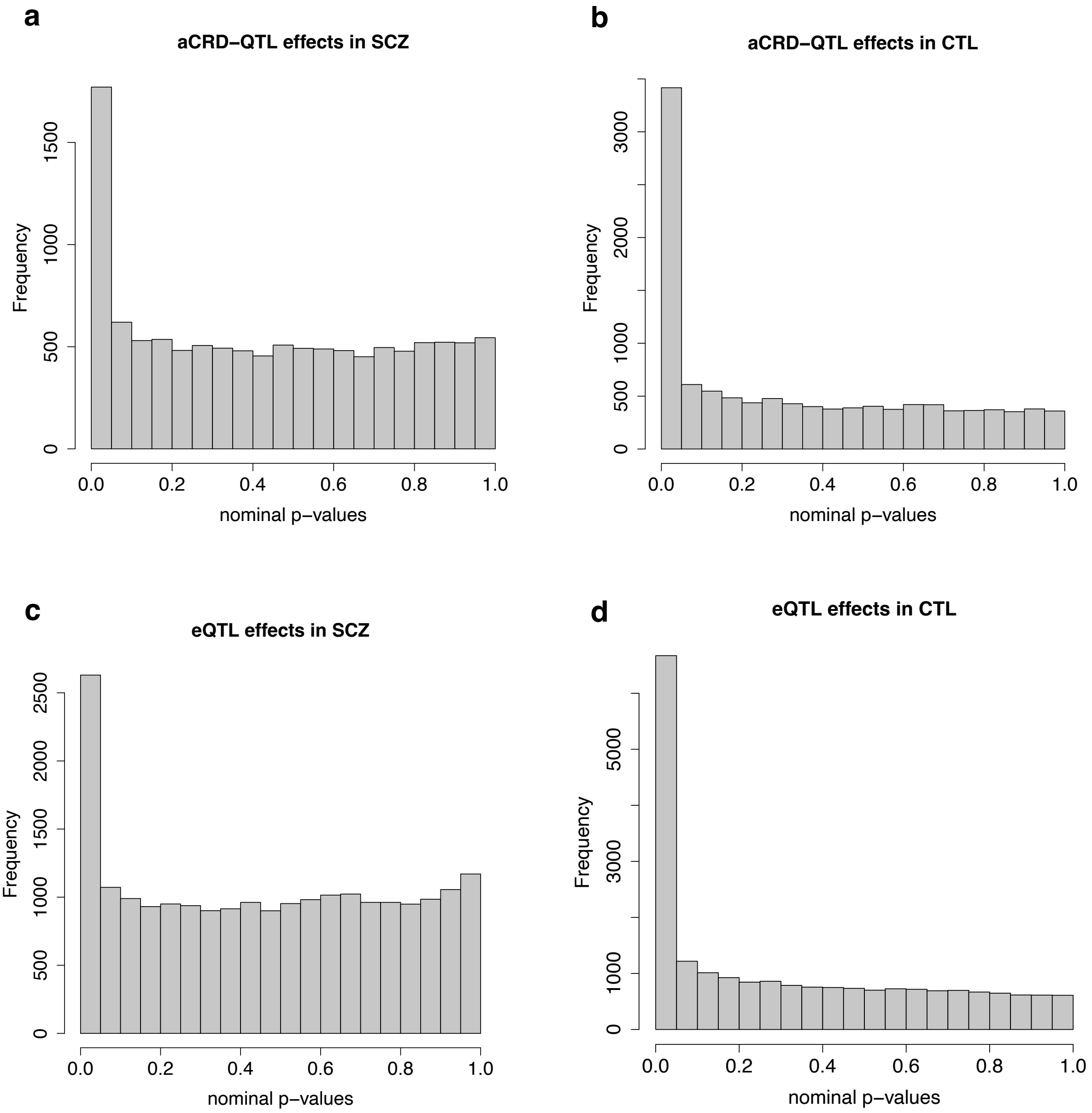

Supplementary Fig. 17. QTL discovery. P-value distribution for QTL and CRD activity/gene expression associations (a,c) in SCZ cases and (b,d) in controls. At 5% FDR and in cis, 857 and 3,144 functionally independent aCRD-QTLs, and 987 and 6,716 functionally independent eQTLs in SCZ cases and controls, respectively, were discovered.

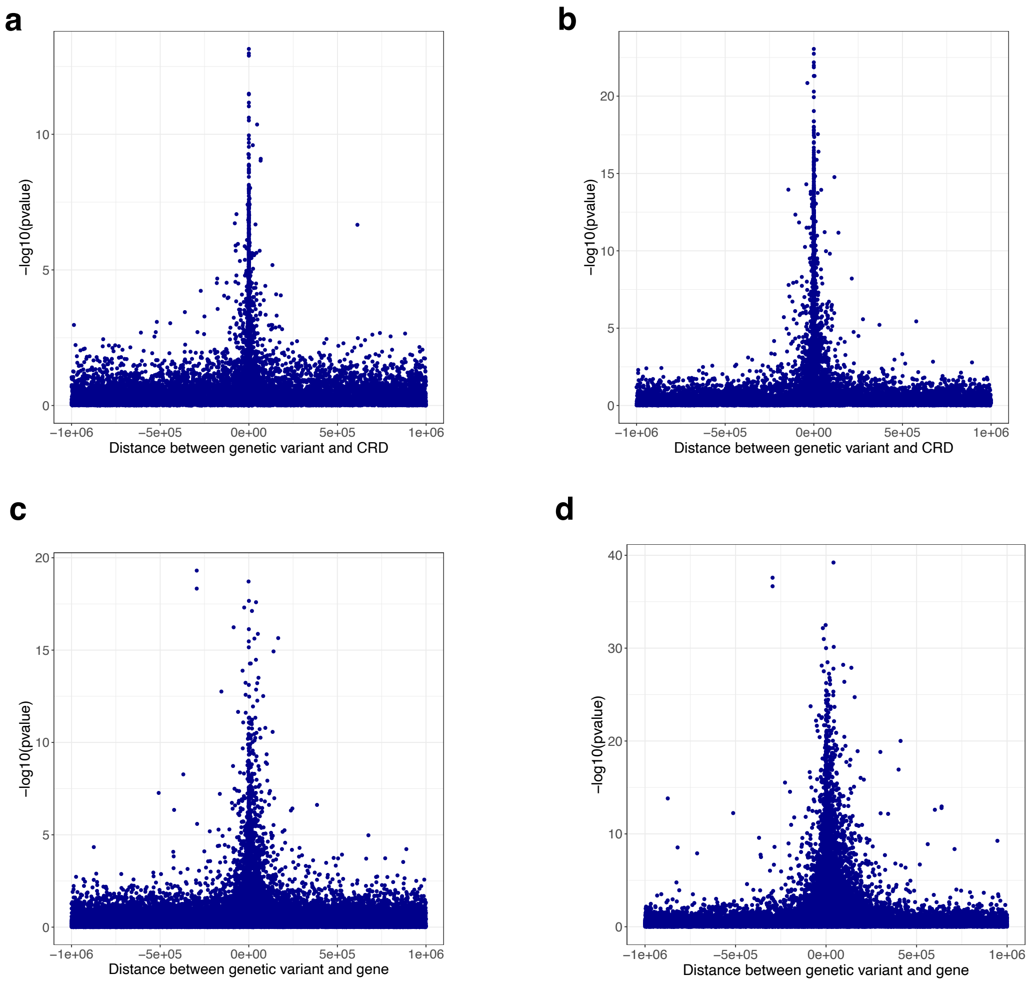

Supplementary Fig. 18. CRD/gene distance in base pairs from associated QTL. Genomic distance between genetic variant and CRD/gene as a function of the strength of association given in -log10 p-values (a,c) for SCZ cases and (b,d) for controls.

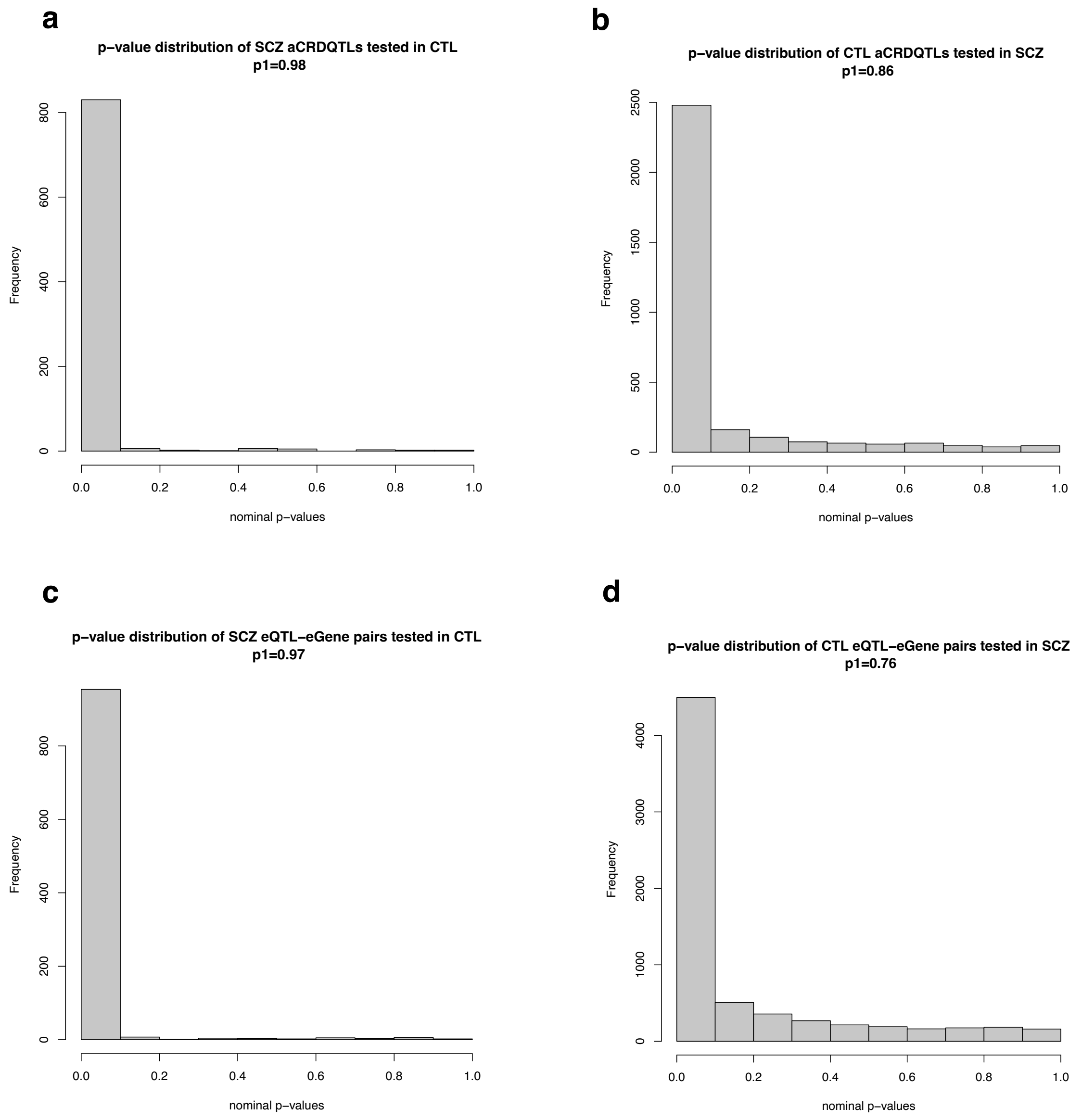

Supplementary Fig. 19. Proportion of sharing aCRD-QTL and eQTL effects between SCZ cases and controls based on π1 estimate. P-value distributions of SCZ-identified (a) aCRD-QTL and (c) eQTL effects tested in controls, and control-identified (b) aCRD-QTL and (d) eQTL effects tested in SCZ cases.

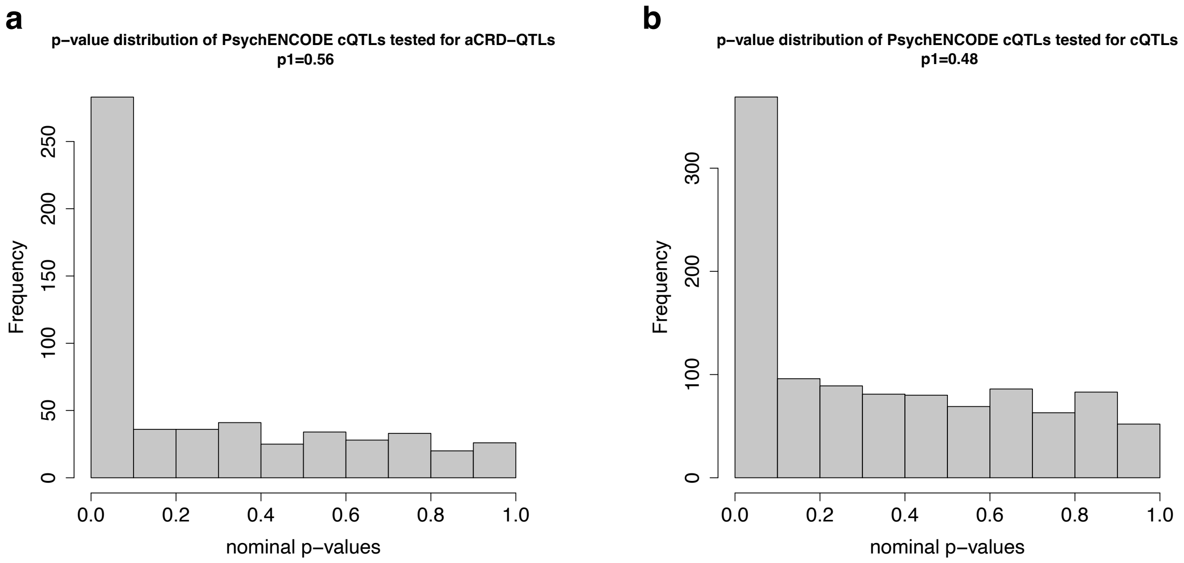

Supplementary Fig. 20. Replication of PsychENCODE cQTLs for aCRD-QTLs and cQTLs. Proportion of sharing based on $\pi 1$ between 2,279 QTLs for H3K27ac peaks detected at FDR 5% in PsychENCODE in relation to a) aCRD-QTL and b) cQTL signals identified across SCZ cases and controls in the current study.

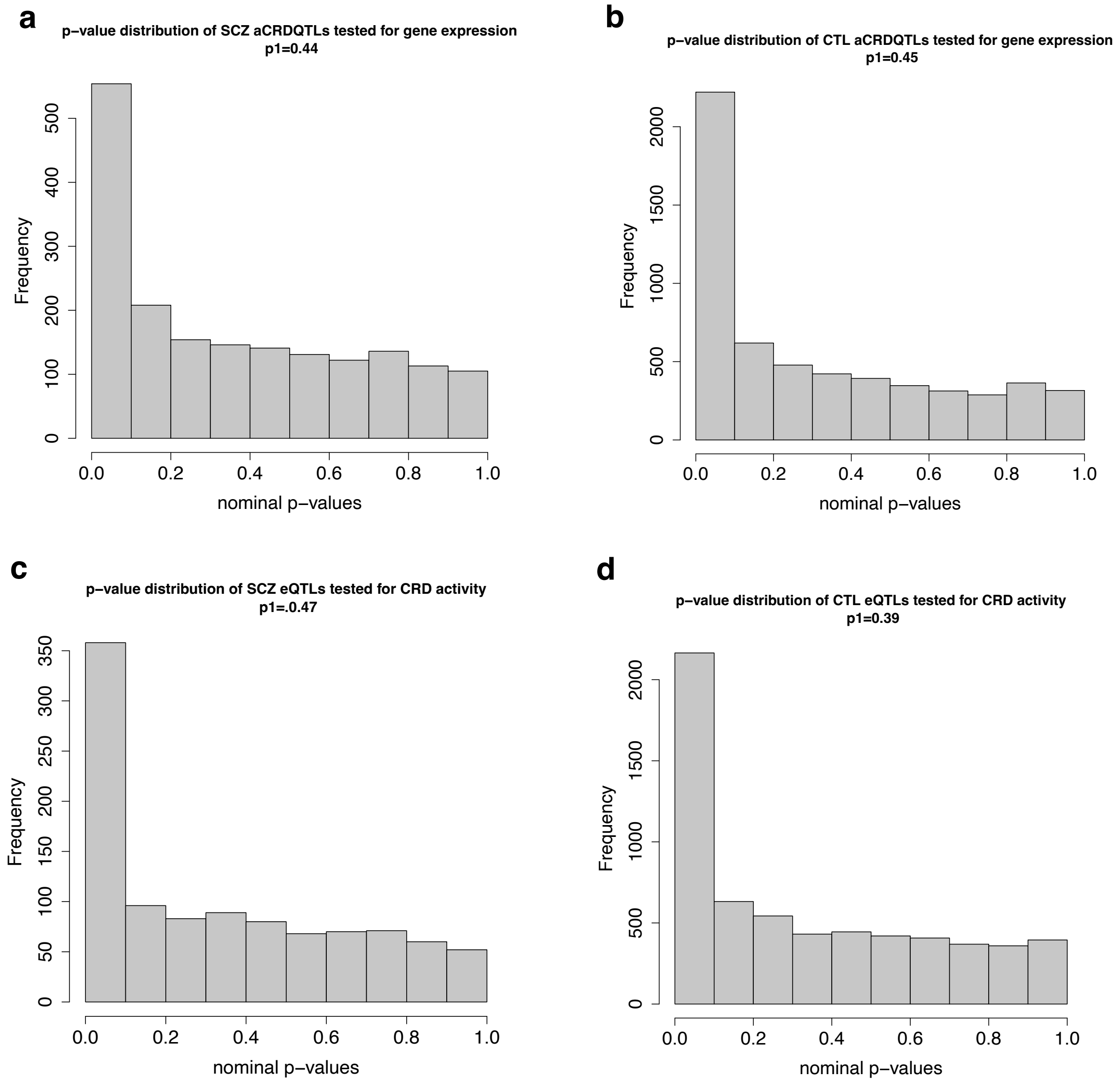

Supplementary Fig. 21. Proportion of sharing aCRD-QTL and eQTL effects for gene expression and for CRD activity, respectively, based on π1 estimate. P-value distribution of (a) SCZ-identified and (b) controls-identified aCRD-QTLs tested for gene expression over CRD-gene associations identified at nominal significance level. P-value distribution of (c) SCZ-identified and (d) controls-identified eQTLs tested for CRD activity over gene-CRD associations identified at nominal significance level.

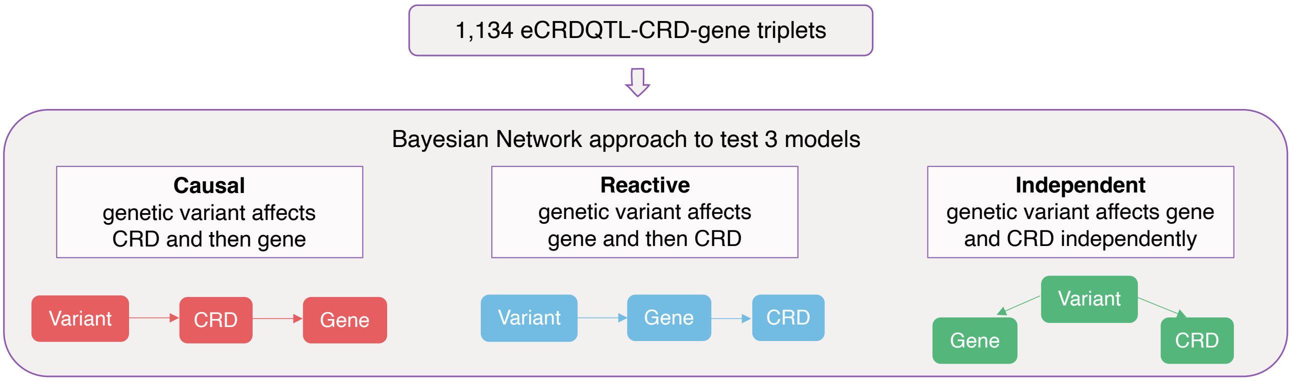

Supplementary Fig. 22. Bayesian Network approach. Schematic of models considered in Bayesian Networks to infer the most likely causal relationship for eCRDQTL-CRD-gene triplets in SCZ cases and controls. eCRD-QTL denotes a genetic variant that affects the activity of a CRD and the expression of a gene that show a significant association with each other.

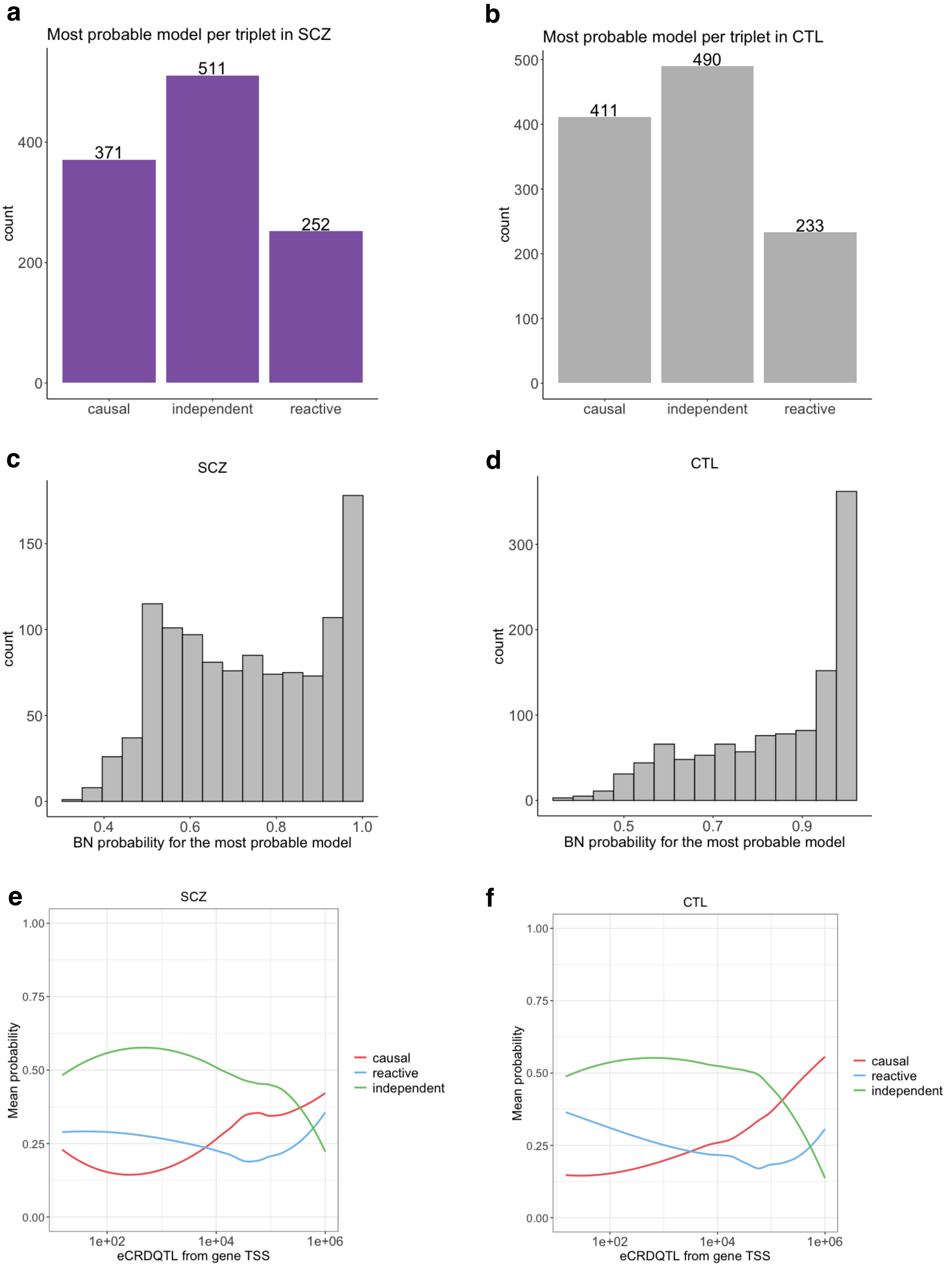

Supplementary Fig. 23. Causal inference estimation for eCRDQTL-CRD-gene triplets. Counts of the most probable model for each triplet (n=1,134) for (a) SCZ cases and (b) controls. Distribution of the probabilities for the most probable model for each triplet for (c) SCZ cases and (d) controls. Mean probabilities for each model as a function of the distance in base pairs between eCRD-QTL and the target gene for (e) SCZ cases and (f) for controls.

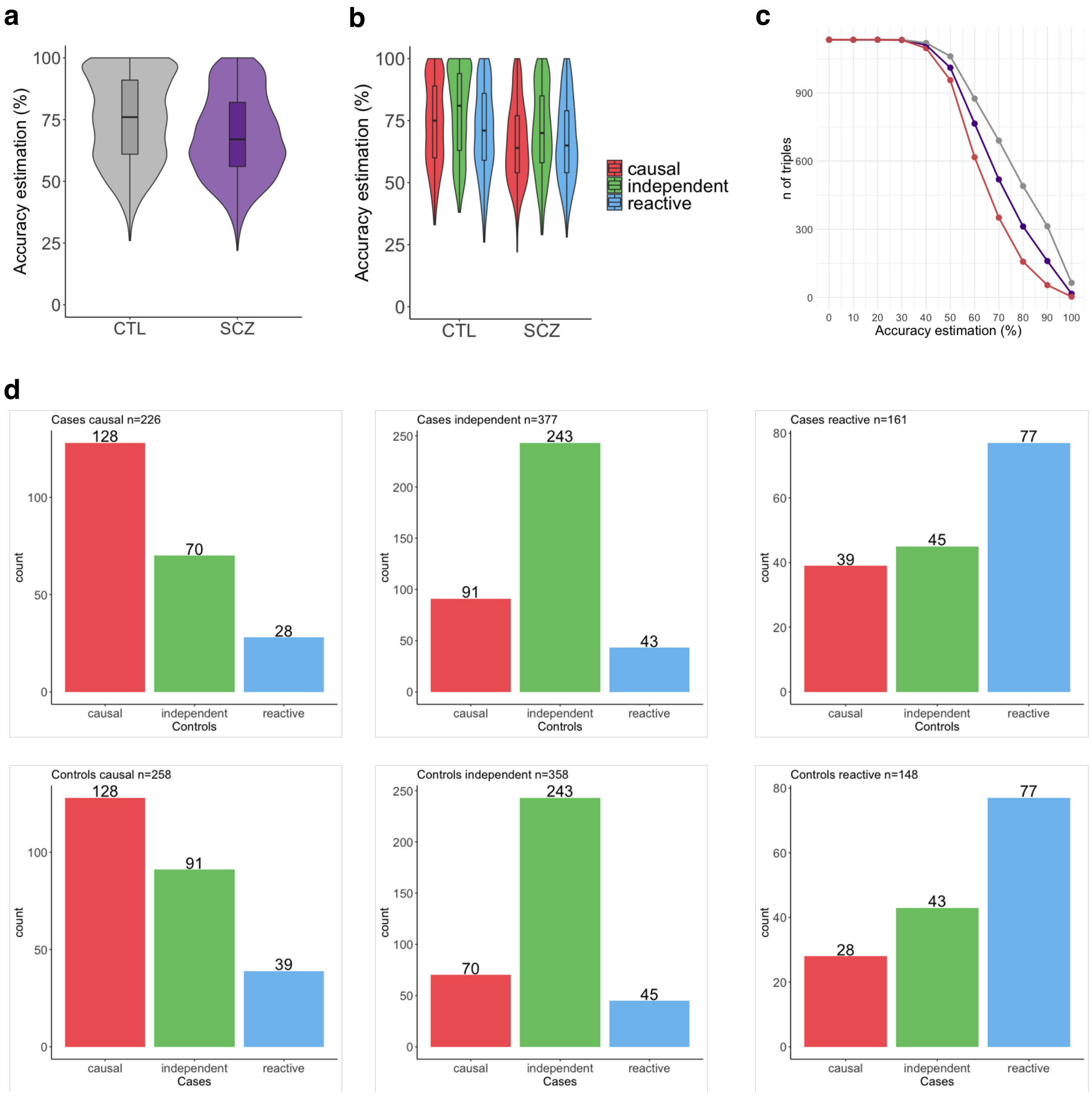

Supplementary Fig. 24. Regulatory mechanism for eCRDQTL-CRD-gene triplets in SCZ cases and controls. Distribution of accuracy estimation (%) denoting how often the most probable model across bootstrapping runs for each triplet was the same as in the original Bayesian Network analysis for SCZ cases and controls (a) across models and (b) by model. (c) Triplet counts as a function of accuracy estimation; purple colour denotes triplet counts for SCZ cases, grey for controls and red indicates triplet counts at the intersect of accuracy estimation for SCZ cases and controls. (d) Comparison of the direction of effect from eQTL-CRD onto molecular phenotypes between SCZ cases and controls for 764 triplets that surpassed the accuracy estimation of 55%.

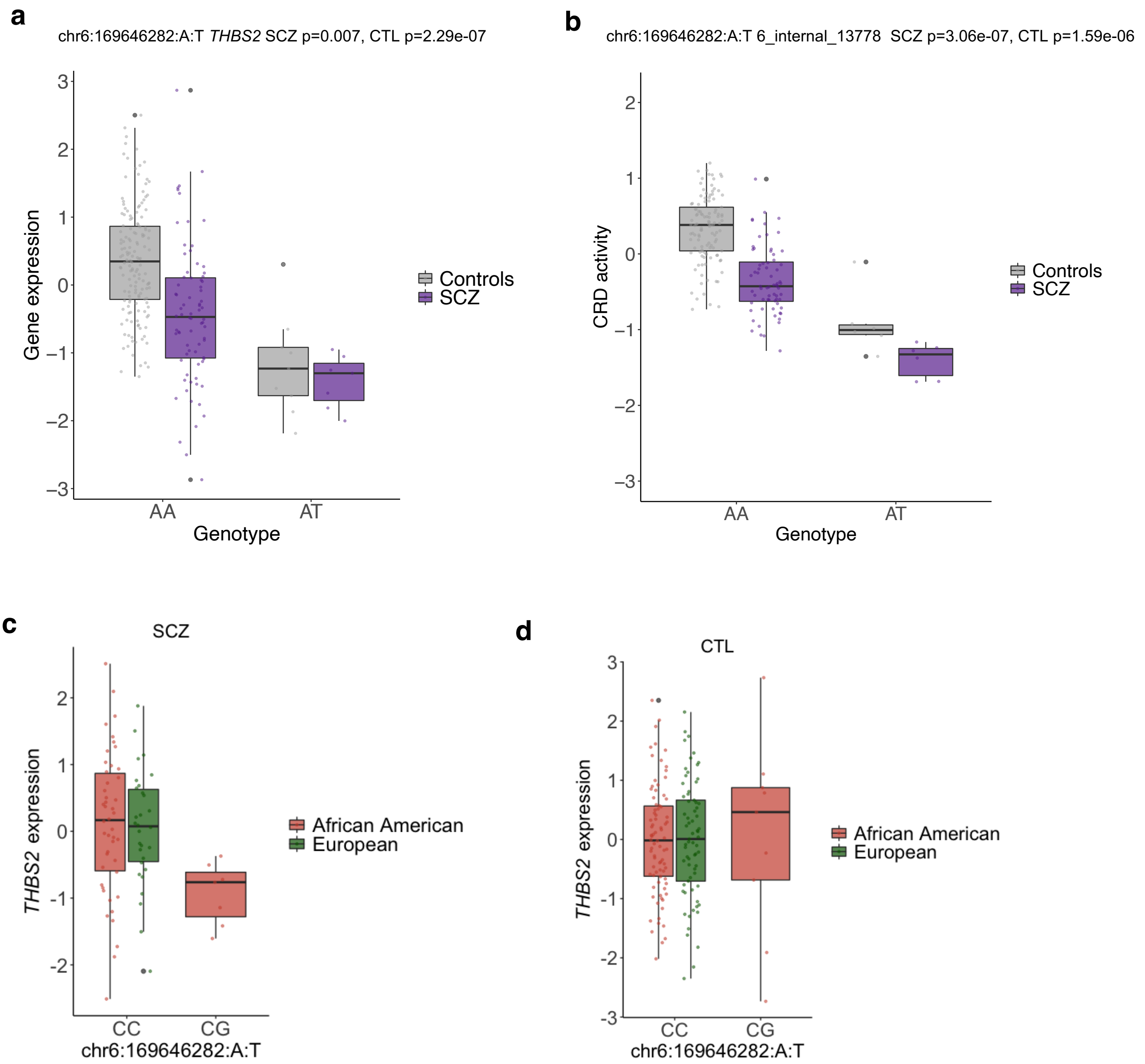

Supplementary Fig. 25. Example of the mechanistic change in the regulation of gene expression between SCZ cases and controls. The triplet consists of an eCRD-QTL chr6:169646282:A:T, gene *THBS2* and a CRD composed of 18 REs on chr6:169541739-169999929. (a,b) Genotype-dependent effect for eCRD-QTL chr6:169646282:A:T on *THBS2* expression and CRD activity. (c,d) Genotype-dependent effect for eCRD-QTL chr6:169646282:A:T on *THBS2* expression by ancestral group for SCZ cases and for controls (CTL).

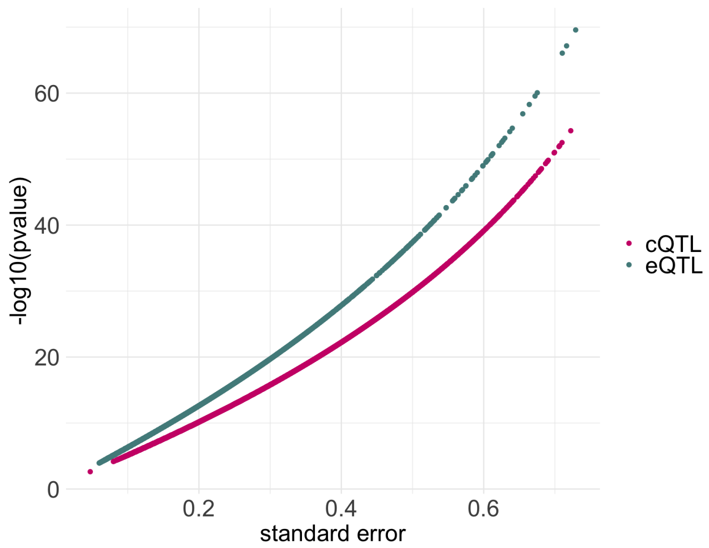

Supplementary Fig. 26. Standard error as a function of significance value for cQTL variants detected for H3K27ac peaks and for eQTL variants detected for gene expression across all samples.
